# Carboxy-Amidated AamAP1-Lys has Superior Conformational Flexibility and Accelerated Killing of Gram-Negative Bacteria

**DOI:** 10.1021/acs.biochem.4c00580

**Published:** 2025-01-28

**Authors:** Rosalind J. Van Wyk, June C. Serem, Carel B. Oosthuizen, Dorothy Semenya, Miruna Serian, Christian D. Lorenz, A. James Mason, Megan J. Bester, Anabella R. M. Gaspar

**Affiliations:** † Department of Biochemistry, Genetics and Microbiology, Faculty of Natural and Agricultural Sciences, University of Pretoria, Pretoria 0002, South Africa; ‡ Department of Anatomy, Faculty of Health Sciences, University of Pretoria, Pretoria 0002, South Africa; § Drug Discovery and Development Centre (H3D), University of Cape Town, Cape Town 7701, South Africa; ∥ Institute of Pharmaceutical Science, School of Cancer & Pharmaceutical Sciences, King’s College London, London SE1 9NH, United Kingdom; ⊥ Department of Physics, Faculty of Natural, Mathematical and Engineering Sciences, King’s College London, London WC2R 2LS, United Kingdom.; # Department of Engineering, Faculty of Natural, Mathematical and Engineering Sciences, King’s College London, London WC2R 2LS, United Kingdom

## Abstract

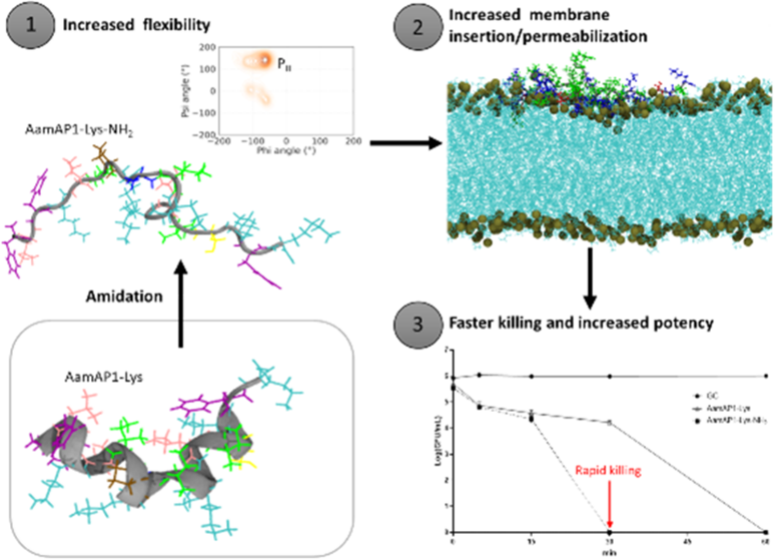

C-terminal amidation of antimicrobial peptides (AMPs) is a frequent minor modification used to improve antibacterial potency, commonly ascribed to increased positive charge, protection from proteases, and a stabilized secondary structure. Although the activity of AMPs is primarily associated with the ability to penetrate bacterial membranes, hitherto the effect of amidation on this interaction has not been understood in detail. Here, we show that amidation of the scorpion-derived membranolytic peptide AamAP1-Lys produces a potent analog with faster bactericidal activity, increased membrane permeabilization, and greater Gram-negative membrane penetration associated with greater conformational flexibility. AamAP1-lys-NH_2_ has improved antibiofilm activity against *Acinetobacter baumannii* and *Escherichia coli*, benefits from a two- to 3-fold selectivity improvement, and provides protection against *A. baumannii* infection in a *Galleria mellonella* burn wound model. Circular dichroism spectroscopy shows both peptides adopt α-helix conformations in the steady state. However, molecular dynamics (MD) simulations reveal that, during initial binding, AamAP1-Lys-NH_2_ has greater conformation heterogeneity, with substantial polyproline-II conformation detected alongside α-helix, and penetrates the bilayer more readily than AamAP1-Lys. AamAP1-Lys-NH_2_ induced membrane permeabilization of *A. baumannii* occurs only above a critical concentration with slow and weak permeabilization and slow killing occurring at its lower MIC but causes greater and faster permeabilization than AamAP1-Lys, and kills more rapidly, when applied at equal concentrations. Therefore, while the increased potency of AamAP1-Lys-NH_2_ is associated with slow bactericidal killing, amidation, and the conformational flexibility it induces, affords an improvement in the AMP pharmacodynamic profile and may need to be considered to achieve improved therapeutic performance.

## Introduction

In the current post-antibiotic era, the rise in antimicrobial resistant (AMR) infections not only causes high rates of morbidity and mortality in humans but also puts the food and economic sectors at risk.^1,2^ Of greatest concern are infections caused by multidrug resistant (MDR) Gram-negative bacteria such as *Pseudomonas aeruginosa*, *Acinetobacter baumannii* and *Enterobacteriaceae* (*Klebsiella pneumoniae*, *Escherichia coli* and *Enterobacter* spp.).^3,4^ Consequently these bacteria are listed by the World Health Organization (WHO) as critical or high priority pathogens where there is an urgent unmet need for alternative therapeutic agents.^5^ Challenges associated with MDR infections are the resistance of these pathogens to three or more antibiotics and tendency to form difficult to eradicate biofilms in human tissues and on medical devices and equipment.^6−8^ The development of alternative antimicrobial agents, with novel modes of action and reduced risk for resistance development is of the utmost importance if we are to combat the rise in MDR and extensively drug resistant (XDR) Gram-negative infections, especially nosocomial infections caused by *A. baumannii*.^3,9^

Antimicrobial peptides (AMPs) are potential drug candidates for the treatment of such infections and are small (<50 amino acids), generally cationic and amphipathic but with diverse secondary structures and modes of action. AMPs commonly target the negatively charged cell membranes of bacteria through electrostatic and hydrophobic interactions, followed by nonspecific membrane permeabilization and/or penetration.^10^ Many AMPs also have intracellular targets and act either by directly binding or disrupting the biosynthesis of the cell wall, DNA, RNA and proteins.^11,12^ The nonspecific and multifaceted modes of action of AMPs provide them with a broad-spectrum of activity and reduced risk for resistance development and distinguishes AMPs as important leads for the development of the next generation of antibiotics.^13^ Even though many AMPs show great activity against Gram-negative pathogens, their successful translation to clinical application is often halted due to unwanted side effects related to cytotoxicity and low specificity as well as short circulating half-life and instability in physiological conditions.^14,15^

To address the unsuccessful translation of AMPs to the market, various design strategies have been explored.^10,16^ Major modifications include peptide cyclization and lipidation which can enhance stability and resistance to degradation but more conservative modifications including residue substitutions, deletions, end-tagging or N/C-terminal modifications can enhance activity, stability and selectivity.^16^ The structure and properties of these peptides are intricately linked to their mode of action against bacteria. Minor changes can alter their interaction with bacterial membranes, thereby affecting their antimicrobial activity, consequently even small modifications can have significant impacts on the efficacy of AMPs.^17,18^

By carefully designing and modifying AMPs, the aim is to strike a balance between antimicrobial efficacy and safety by minimizing toxicity while maximizing potency.^19^ Peptide AamAP1, with sequence FLFSLIPHAIGGLISAFK (charge +1), was originally isolated from the North African scorpion *Androctonus amoreuxii*. Several amino acid residues (Ser4, His8, Gly11, Gly12, and Ala16) of AamAP1 was substituted with Lys to generate the membranolytic derivative AamAP1-Lys. The rationale behind these modifications was to enhance the positive charge and optimize other key physicochemical properties of AamAP1. The resulting sequence of AamAP1-Lys, FLFKLIPKAIKKLISKFK, increases the net charge to +6, reduces the hydrophobicity, and enhances its α-helicity compared to the parent peptide AamAP1. These modifications led to a 10-fold increase in antimicrobial activity, mild cytotoxicity to eukaryotic cells, and an improved selectivity index.^20,21^

In our own previous work,^19^ AamAP1-Lys was amidated at its C-terminal, which is a simple post-translational modification present in many native bioactive peptides and commonly used in the field to improve AMP potency.^22^ C-terminal amidation resulted in an analog, AamAP1-Lys-NH_2_, with a further 2-fold increase in antibacterial activity against a panel of susceptible and antibiotic resistant Gram-positive and Gram-negative bacteria.^19^ Additionally, amidation reduced the hydrophobicity of the peptide, as demonstrated by a decrease in the elution time of AamAP1-Lys from 45.5 to 43 min on a C18 high-performance liquid chromatography (HPLC) column.^19^

One of the roles of C-terminal amidation, in both native and synthetic peptides, is to offer protection against enzymatic degradation.^23−25^ In addition, C-terminal amidation increases the positive charge of AMPs by introducing a neutral CONH_2_ group. An increase in positive charge leads to enhanced electrostatic binding affinity of AMPs for negatively charged bacterial membranes^26^ and can thus improve the antibacterial activity of amidated AMPs. However, it has been argued that charge alone cannot explain the improved activity of amidated AMPs.^27^ More important might be a change in the stability of the secondary structure that is implicated in the improved membrane interaction of many amidated AMPs.^28−30^ More subtle effects due to C-terminal amidation have been detected by molecular dynamics (MD) simulations. These include changes in the peptide orientation when binding and inserting into membranes,^29,31−33^ as well as leading to pore formation as in the case for an amidated anoplin derivative.^34^

Recently we have applied all atom MD simulation in conjunction with a range of *in vitro* biophysical studies to enable an appreciation of how relatively modest changes in AMP sequence affect antibacterial potency and also mechanism of action.^18,35−37^ Here we develop the approach further to better explain the structure–function activity of AamAP1-Lys amidation against Gram-negative pathogens. First, we characterize the effect of AamAP1-Lys amidation on selectivity toward Gram-negative bacteria, antibiofilm activity and the ability to protect *Galleria mellonella* larvae from infection with a resistant clinical strain of *A. baumannii*. We then study mechanistic aspects of the antibacterial activity, using MD simulations to investigate the effect of C-terminal amidation on peptide conformation, self-association and penetration in models of the Gram-negative bacteria plasma membrane and relate this to greater membrane permeabilization and faster *in vitro* bactericidal activity against *A. baumannii*.

## Materials and Methods

### Peptides

Crude samples of AamAP1-Lys (with sequence FLFKLIPKAIKKLISKFK) and AamAP1-Lys-NH_2_ (with sequence FLFKLIPKAIKKLISKFK-NH_2_) were obtained from Cambridge Research Biochemicals (Cleveland, UK) and purified using preparative reverse-phase high-performance liquid chromatography (RP-HPLC) to >95% purity. To achieve elution, a water/acetonitrile gradient (solvent A; 0.1% trifluoroacetic acid (TFA) in water (v/v), solvent B; 0.1% TFA in 100% acetonitrile (ACN)(v/v)) at a flow rate of 8 mL/min was used as described previously.^19^ The collected fractions were spun in a speed-vac to remove ACN followed by freeze-drying for 24 h. The lyophilized peptides were dissolved in 10% (v/v) acetic acid and freeze-dried for a second time to remove residual TFA. The lyophilized peptides were weighed and stored in low binding Eppendorf tubes until used. Melittin (>95% pure), was obtained from GenScript (Piscataway NJ, USA) and used as the positive control in the inner membrane permeability assays.

### Circular Dichroism Spectroscopy

Peptide secondary structure was determined in Tris buffer, a sodium dodecyl sulfate (SDS) solution and following binding to small unilamellar vesicles (SUVs) according to the method adapted from Manzo et al.^36^ The SUVs were prepared by solubilizing the lipids 1-palmitoyl-2-oleoyl-*sn*-glycero-3-[phosphor-rac-(1-glycerol)] (POPG) and 1-palmitoyl-2-oleoyl-*sn*-glycero-3-phosphoethanolamine (POPE) (Avanti Polar Lipids, Inc., Alabama, USA) (POPE/POPG, 75:25, mol/mol) in 1 mL chloroform (Sigma-Aldrich, St Louis, USA) and then dried with rotary-evaporation. The lipid films were kept overnight under vacuum to remove any residual organic solvent, suspended in 5 mL of 5 mM Tris buffer (pH 7.0) and then subjected to five rapid freeze–thaw cycles (using liquid nitrogen and a water bath at 40 °C). Subsequently, the lipid suspensions were sonicated (2 × 5 min) on a Soniprep 150 (Measuring and Scientific Equipment, London, UK) on ice. All SUVs samples were stored for a maximum of 5 days at 4 °C before use. Peptides of 50 μM were prepared in 5 mM Tris (pH 7.0), 50 mM SDS in 5 mM Tris (pH 7.0) or 5 mM SUV in 5 mM Tris (pH 7.0). The far-UV CD spectra were recorded from 180 nm–260 nm at 23 °C, with a scanning speed of 200 nm/min, a pitch of 0.1 nm and a bandwidth of 2 nm (Jasco J-810 spectropolarimeter, Ishikawa-machi, Tokyo, Japan). The blank was a solution without peptide. The average of 10 scans, repeated twice, was used to generate the raw data. The mean residue molar ellipticity [θ] was calculated as
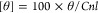
1where *C* is the peptide concentration in mg/mL, *n* is the number of residues, *l* is the path length and θ is the signal measurement in millidegrees.

### MD Simulations and Analysis

All MD simulations were carried out using methodology described by Manzo et al.^36,37^ employing Gromacs 2020.1^38^ and the CHARMM36 all-atom force field.^39,40^ The simulated lipid bilayers consisted of 256 total lipids (128 per leaflet) and were built using CHARMM-GUI.^41^ The model Gram-negative bacterial plasma membrane^42^ was represented by lipid compositions of POPE/POPG (96 POPE lipids and 32 POPG lipids per leaflet, 3:1). The preliminary starting structures of the peptides were predicted by using the AlphaFold2 system developed by DeepMind.^43^ Simulations of these structures were then run in water for 1 μs and used as the starting structure of the peptide inserted above the Gram-negative model membrane. Four peptides were inserted at 8 Å above the membranes in each quadrant of the membrane. The solvation, energy minimization and equilibration of the simulation systems were performed as described.^37^ Briefly, the membrane and the peptides were solved by an aqueous environment containing 150 mM sodium chloride (NaCl). The CHARMM-modified TIP3P model was used to describe the water, while the CHARMM36 force field was used to describe the ions. Then an energy minimization using the steepest descent algorithm was performed on the resulting system. The canonical NVT and isothermal–isobaric NPT ensembles were used to carry out equilibration for 100 ps and 1 ns, respectively. In the NVT ensemble, number of atoms (N), volume (V) and temperature (T) are kept constant. In the NPT ensemble, N, pressure (P) and T are kept constant. The production simulations were run for 200 ns, trajectories recorded at 2 fs intervals. Data analysis was conducted using python scripts which were developed in-house at King’s College (London, UK) with MDAnalysis 2.7.0 (http://mdanalysis.org) and Python 3.7.0.^44,45^ To analyze the insertion of the peptides into the Gram-negative model membranes, *Z*-position analysis was done. The LeafletFinder MDAnalysis module^45^ was used to determine the average *z*-position of the phosphorus (P) atoms within the upper leaflet of the bilayer. The *z*-position of the α-carbons in each residue was determined at each time step in the trajectory and averaged across the 4 peptides inserted. The mean *z*-positions of the residue α-carbons were subtracted from the mean *z*-position of the upper leaflet phosphate atoms to determine the mean relative *z*-position (nm) of the α-carbons of the peptide residues from the phosphorus atoms, over the duration of the simulation. The relative *z*-positions of the α-carbons in the residues are presented as heatmaps. The HydrogenBondAnalysis MDAnalysis module^45^ was used to determine total number of hydrogen bonds between the peptide residues and lipid headgroups of the bilayers. The guess_acceptors and guess_hydrogens class methods were used to create atom selections. Hydrogen bonds were identified via the following geometric criteria: (1) the donor–acceptor distance (rDA) must be less than cutoff of distance of 3 Å and (2) the donor-hydrogen-acceptor angle (θDHA) must be greater than a cutoff of 150°. The average total hydrogen bonds formed by each peptide residue and the lipid headgroups over the duration of the simulation in intervals of 10 steps are presented as box-and-whisker plots. The MDAnalysis.analysis.dihedrals module and the Dihedral class was used to calculate the psi and phi dihedral angles for each residue of the peptides over the first (0–20 ns) and last (180–200 ns) part of the simulations. The clustering of the psi and phi dihedral angles of the four peptides over the first and last 20 ns of the simulation or for each individual peptide over the last 80 ns were presented as Ramachandran contour plots. The circular variance was calculated using a function which returns an average and standard deviation of a set of angles taking into account that angles are a circular quantity. Circular variance is a value between 0 and 1 and not given in degrees. The largest aggregate formed during each time step during each simulation was determined using networkX^46^ to find connectivity and the MDAnalysis.analysis.distances module^45^ was used to set a cutoff distance of 6 Å. The size of the aggregates as well as the distances between the residues implicated in the aggregates are presented as heatmaps. The secondary structures and interactions of the peptides in the Gram-negative model membranes were visually presented using Visual Molecular Dynamics (VMD) software 1.9.4.^47^

### Minimum Inhibitory Concentration (MIC) Assay

The susceptible Gram-negative bacterial strains were obtained from the American Type Culture Collection (ATCC). The *A. baumannii* clinical isolates were obtained from H3D, Cape Town, SA. Briefly, bacteria were streaked out onto Tryptic Soy Broth (TSB) agar plates and incubated overnight at 37 °C. The next day, 3–5 colonies were picked and resuspended in 4 mL Mueller Hinton Broth (MHB) (Merck, Darmstadt, Germany) to an OD_600nm_ of 0.1. The bacterial inoculum was further diluted 100× to an OD_600nm_ of 0.001 to yield approximately 2 × 10^6^ CFU/mL. Stock peptide solutions of 5 mM were prepared in sterile ddH_2_O. Serial 2× dilutions of the peptides were prepared in a polypropylene 96 well plate (Thermo Fisher Scientific, Roskilde, Denmark) with a final volume of 50 μL. Subsequently, 50 μL of the prepared bacterial suspension was added to each well to obtain a final bacterial cell density of 1 × 10^6^ CFU/mL with a peptide concentration range of 0.125 to 64 μM. The negative control was bacteria in MHB, the positive control was 128 μg/mL polymyxin B (Merck, Darmstadt, Germany) and the sterility control was MHB without bacteria. The plate was incubated statically for 18–24 h at 37 °C. The MIC was determined visually and was defined as the concentration at which no visible bacterial growth was observed. Experiments were performed in triplicate, with each peptide tested in duplicate (*n* = 6).

### Cytotoxicity and Selectivity

For cytotoxicity screening against human keratinocytes (HaCat) cells, the peptide stock solutions were diluted in 1% dimethyl sulfoxide (DMSO) (Sigma-Aldrich, St. Louis, USA) and 10 μL was transferred to the wells of a NEST 96-well microtiter plate (Whitehead Scientific, Modderfontein, Gauteng, SA), containing 24 h cultures of HaCat cells initially plated at 1.1 × 10^5^ cells/well in 90 μL Dulbecco’s Modified Eagles Medium (DMEM) containing 10% fetal calf serum (FCS). The final peptide concentration range was 4 to 1024 μg/mL. The positive control was cells treated with 0.1% Triton-X100, the negative controls were cells only, and the sterility control was media only. After incubation for 21 h in 90% humidity, 5% CO_2_ at 37 °C, 11 μL of 3-(4,5-dimethylthiazol-2-yl)-2,5-diphenyltetrazolium bromide (MTT) reagent (1 mg/mL in 1 M PBS) was added to each well. After a further 3 h incubation, the media was removed, the plates were dried, and the formazan crystals were solubilized with 50 μL of a 25% DMSO in ethanol (v/v) solution. The absorbance was measured at 570 nm with a FLUOstar Omega spectrophotometer (BMG Labtech, Germany).

For cytotoxicity screening against the hepatocyte carcinoma (HepG2) cells, the peptide samples were prepared to a 10 mM stock solution in 100% DMSO. Further dilutions to the desired starting concentration were freshly prepared in DMEM containing 10% FCS at the start of the experiment. HepG2 cells were plated to a density of 1.0 × 10^5^ cells/well in 96-well plates and allowed to attach for 24 h. Subsequently, peptides were added at various concentrations from 50 μM down to 16 nM and the cells incubated for a further 48 h. Emetine was used as the positive control, since it shows nonspecific cytotoxicity to mammalian cells. After 44 h, MTT was added as described above and the plates and after an additional 4 h at 37°, the plates were centrifuged at 200 rpm for 5 min to pellet the reduced dye crystals. The growth medium was carefully removed and 50 μL of DMSO added and the plate was then gently shaken to ensure complete dissolution. The absorbance was then measured at 540 nm. Cell viability of HaCat and HepG2 cells was plotted against peptide concentration and the LC_50_ values were obtained using a nonlinear dose–response curve fitting. Experiments were performed in triplicate and each peptide tested in duplicate (*n* = 6). The LC_50_ was defined as the lethal peptide concentration at which 50% of the cells were viable relative to the negative control. The selectivity was determined by ratios of LC_50_/MIC and was defined as selectivity indices (SIs).^48^

### Antibiofilm Assays

The minimum biofilm prevention concentration (MBPC), minimum biofilm eradication concentration (MBEC) and minimum biofilm inhibitory concentration (MBIC) of the peptides were determined using an adapted biofilm susceptibility assay described by Moskowitz et al.^49^ The MBEC and MBIC assays are performed using 24 h established biofilms and are parameters used to investigate the eradication and inhibition of mature biofilms by the peptides. In contrast, the MBPC assay is performed on bacterial cells adhered to a surface after 1 h incubation without treatment and is a parameter that investigates the ability of the peptides to eliminate and prevent early colonization of bacterial cells on surfaces and thus the prevention of mature biofilm formation. Briefly, overnight streak plates of *A. baumannii* NICD 15283 and *Escherichia coli* ATCC 700928 on Luria Broth (LB) (Merck, Darmstadt, Germany) agar plates were prepared. Subsequently, 3–5 colonies from the LB agar plates were inoculated in MHB and diluted to an OD_600nm_ between 0.1 to 0.15 using MHB. To each well of a polystyrene 96-well microtiter flat bottom plate (Thermo Fisher Scientific, Roskilde, Denmark) 100 μL of the bacterial suspension was added to prepare inoculum plates. To determine the MBPC, the peg lid (Nunc Immuno TSP lids from Thermo Fisher Scientific, Roskilde, Denmark) was placed onto the inoculum plate and incubated at 37 °C, stationary, for 1 h to allow some cells to adhere to the peg lid without forming a mature biofilm. Whereas for the MBEC and MBIC determination, the peg lids were placed onto the inoculum plates and incubated for 24 h to allow for mature biofilm formation. Subsequently, treatment plates were prepared by 2-fold serial diluting the peptides in MHB (total volume of 100 μL; concentration range 0.02–2.56 mg/mL) in a polypropylene 96-well microtiter plate. The negative control was bacteria only, the positive control was 128 μg/mL polymyxin B and the sterility control was MHB. The peg lids from the inoculum plates were washed three times in 150 μL sterile water in three separate 96-well wash plates before transferring it to the treatment plates. The treatment plates were incubated for 24 h at 37 °C. Thereafter the peg lids were removed and washed as described above, and were placed in 96 well polystyrene recovery plates containing 100 μL MHB in each well. The recovery plates with the peg lids were covered with parafilm, placed inside a plastic zip lock bag and sonicated for 20 min (*A. baumannii* NICD 15283 biofilms) or 15 min (*E. coli* ATCC 700928 biofilms) to remove the attached remaining biofilms/cells. To determine the MBPC and MBEC, a 6 μL volume from each well of the corresponding recovery plates was spotted on large LB agar plates and incubated overnight at 37 °C. The MBEC or MBPC was defined as the lowest peptide concentration that resulted in no growth from the corresponding spot plate. To determine the MBIC, the OD_650nm_ of each well in the corresponding recovery plate was measured at 0 h and after 5 h of stationary incubation at 37 °C. Adequate biofilm growth for the negative control wells was defined as a mean OD_650nm_ difference (OD_650nm_ at 5 h minus the OD_650nm_ at 0 h) that is ≥0.05. The MBIC was defined as the lowest concentration of peptide that resulted in ≤10% biofilm growth.^49^ The percentage biofilm growth was determined as
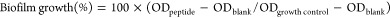
2where OD_peptide_ is the optical density of recovered biofilm exposed to known concentration of peptide, OD_blank_ is the optical density of the media only and OD_growth control_ is the optical density of untreated recovered biofilm. MBPC and MBEC experiments were performed in duplicate with each peptide tested in duplicate (*n* = 4). The MBIC experiments were performed in triplicate with each peptide tested in duplicate (*n* = 6)

### *G. Mellonella* Burn Wound Infection Model

*G. mellonella* (Live Foods UK Ltd.) of similar size, weight and length were selected (200–300 mg). Prior to use, larvae were sorted into Petri dishes (10 larvae per plate) lined with Whatman filter paper (Fisher, UK), and stored at 4 °C until use. The selected *Galleria* were then submerged in a 50% ethanol solution for 20 s to decontaminate. Similar to the previously described method,^50^ a parcel pin nail (Challenge zinc plated 30 mm nail) was heated over a Bunsen burner until red hot. The nail was removed from the flame and allowed to cool for around five to eight seconds before being very lightly pressed on the abdomen surface of the *Galleria* for two seconds to inflict a small light brown superficial burn mark of around of approximately 3–4 mm^2^. Immediately post burn, the wound was inoculated with a single colony of *A. baumannii* ATCC 17978 applied directly to the burn site. Alternatively, or in addition, sterile PBS (5 μL) was applied as a fluid resuscitation control. For therapy, 5 μL peptide solution was applied topically 1 h after infection. Survival was monitored over 96 h. Mortality was recorded as complete melanization of the larval body and complete loss of motility. Two independent experiments were conducted on two different occasions using 10 larvae per treatment/control group (*n* = 20).

### Time-Kill Kinetics Assay

Treatment plates as described for the MIC assay were prepared. The time-kill assay was undertaken at the MIC of AamAP1-Lys and AamAP1-Lys-NH_2_ and at 4 μM against *A. baumannii* NICD 15283. A volume of 10 μL at the time points of 0, 5, 15, 30, and 60 min was collected, diluted in PBS and then 7 μL spotted onto TSB agar plates. After 16 h stationary incubation at 37 °C, the colonies were counted and converted to log(CFU/mL), for analysis. Duplicate experiments with two technical repeats were undertaken (*n* = 4).

### Membrane Permeability Assay

A protocol similar to that described in Merlino et al.^51^ was followed. Briefly, an *A. baumannii* NICD 15283 inoculum was prepared, resuspended in 1× PBS and the OD_600nm_ was corrected to 0.1 with PBS to obtain approximately 2 × 10^8^ CFU/mL. A 5 mM SYTOX green (Thermo Fisher Scientific, Roskilde, Denmark) stock solution was diluted in PBS to 4 μM. Starting at the MIC of each peptide, serial 2-fold dilutions were prepared in PBS in a black 96-well flat bottom microtiter plate (Thermo Fisher Scientific, Roskilde, Denmark). A 1:1 ratio of 4 μM SYTOX green solution in PBS and the bacterial inoculum were transferred to the black 96-well flat bottom microtiter plates. The final concentrations were 1 μM SYTOX green, approximately 5 × 10^7^ CFU/mL bacteria and peptide concentration ranges of MIC to 1/8 MIC. The negative control was bacteria stained with 1 μM SYTOX green and the positive control was bacteria treated with 4 μM melittin. The relative fluorescence units (RFU) were measured at excitation and emission wavelengths of 485/535 nm (SpectraMax Paradigm spectrophotometer, Molecular Devices LLC, San Jose, USA). Data acquisition was at 37 °C in intervals of 2 min for 60 min. Experiments were performed in triplicate each with duplicate repeats (*n* = 6).

### Data and Statistical Analysis

All data analysis was performed using GraphPad Prism V 7.0 software (San Diego, CA, USA). Nonlinear regressions were performed and a sigmoidal curve with variable slope (Hill slope less than 7) were fitted to the dose response data with constraints set to 100 (Top) and 0 (Bottom).

## Results

### Amidation Improves Antibacterial Potency

The antibacterial potency of AamAP1-Lys and AamAP1-Lys-NH_2_ against planktonic cells was tested against a panel of Gram-negative bacterial pathogens as well as against a wider panel of *A. baumannii* clinical isolates (Table 1). Five of the six *A. baumannii* strains in this panel are clinical isolates with three strains (NICD 15207, NICD 15282, and NICD 15408) being multidrug resistant to ciprofloxacin and Meropenem (MIC ≥ 64 μg/mL) and moxifloxacin (MICs ≥ 32 μg/mL) (Table S2). In addition, a laboratory strain (NCTC 13302), was included in this panel which is also multidrug-resistant to ciprofloxacin and Meropenem (MIC > 64 μg/mL) and moxifloxacin (MIC = 32 μg/mL). The remaining clinical isolate (NICD 15283) is resistant to moxifloxacin (MIC = 32 μg/mL). Both peptides are effective against the panel of Gram-negative bacteria (Table 1), including the antibiotic-resistant clinical isolates. Compared with AamAP1-Lys, the carboxy-amidated peptide is 4-fold more active against the susceptible strains (MIC ranging from 4–35 μg/mL), except for *E. coli* ATCC 700928 for which the activity is the same (MIC 4 μg/mL).

**Table 1:** Antibacterial Activity of AamAP1-Lys and AamAP1-Lys-NH_2_ against a Panel of Susceptible, Resistant, and Clinical Bacterial Strains^a^

	modal MIC [μg/mL] (μM)^b^		
bacteria	AamAP1-Lys	AamAP1-Lys-NH_2_	fold decrease in MIC
*E. coli* ATCC 700928	4 (2)	4 (2)	0
*E. coli* ATCC 25922	35 (16)	9 (4)	4
*P. aeruginosa* ATCC 27853	138 (64)	35 (16)	4
*A. baumannii* ATCC 19606	17 (8)	4 (2)	4
*K. pneumoniae* ATCC BAA-1705	69 (32)	17 (8)	4

	modal MIC [μg/mL] (μM)^b^		
resistant/clinical isolates	AamAP1-Lys	AamAP1-Lys-NH_2_	fold decrease in MIC
*A. baumannii* NCTC 13302 (OXA-25)	35 (16)	9 (4)	4
*A. baumannii* NICD 15126 (Mac^S^, Car^S^)	35 (16)	9 (4)	4
*A. baumannii* NICD 15207 (Car^R^)	35 (16)	9 (4)	4
*A. baumannii* NICD 15282 (Car^R^)	17 (8)	9 (4)	2
*A. baumannii* NICD 15283 (Car^R^)	35 (16)	9 (4)	4
*A. baumannii* NICD 15408 (Car^R^, Col^I^)	17 (8)	9 (4)	2

a ATCC, American Type Culture Collection – USA; CarR, carbapenem resistant; CarS; carbapenem sensitive; ColI, colistin intermediate; MacS, macrolide sensitive; MIC, minimum inhibitory concentration; NCTC, National Collection of Type Cultures – UK; NICD, National Institute For Communicable Diseases – RSA; OXA-25, OXA-type β-lactamases carbapenem resistance.

b Experiments were performed in triplicate and each peptide tested in duplicate (*n* = 6).

Likewise, the amidated peptide is 4-fold more active (MIC of 9 μg/mL) than AamAP1-Lys toward most resistant clinical isolates of *A. baumannii* and the resistant laboratory strain (NCTC 13302). The lack of improvement in the antibacterial potency of the amidated analog against *E. coli* ATCC 700928 may be due to the inherent susceptibility to AMPs of this strain, which could differ from that of other *E. coli* strains and bacterial species. Susceptibility to antimicrobials is influenced by a variety of factors, including differences in the production and utilization of metabolites in bacteria, which can affect both the growth of the strain and its response to antimicrobials.^52,53^ Furthermore, variations in the structure of outer membrane lipopolysaccharide (LPS) core regions and sugar compositions between *E. coli* strains can also play a significant role in modulating their susceptibility to AMPs.^54^

### Amidation Improves Selectivity for Pathogens over Mammalian Cells

For therapeutic applications, AMPs need to successfully eradicate infections, without adversely affecting mammalian cell viability or other physiological processes. The SI defined as the ratio of the LC_50_ (HepG2 or HaCat) to the MIC, is an important parameter to determine when considering potential applications. Toxicity was evaluated using the HaCat and the HepG2 cell lines. These represent cellular models for topical and systemic applications, respectively.^55−57^ The LC_50_ for HaCat and HepG2 cells were determined following the 24 and 48 h exposure of the peptides, respectively (Table 2).

**Table 2:** Cytotoxicity of AamAP1-Lys and AamAP1-Lys-NH_2_ toward Human Cell Lines

	LC_50_ ± SD [μg/mL] (μM)^b^	
human cell line	AamAP1-Lys	AamAP1-Lys-NH_2_
HepG2^a^	51.0 ± 0.97 (23.6 ± 0.45)	26.4 ± 4.91 (12.2 ± 2.27)
HaCat^a^	124.3 ± 1.05 (57.5 ± 0.48)	101.3 ± 1.05 (46.9 ± 0.49)

a A 0.1% Triton X solution was used as a positive control to induce 100% cytotoxicity.

b Experiments performed in triplicate and independently repeated twice (*n* = 6).

The incubation times of the HaCat (24 h) and HepG2 (48 h) cells with peptides were chosen to represent a single population doubling respectively for each cell line. Against both cell lines, amidation increases the cytotoxicity of AamAP1-Lys. Furthermore, both peptides have lower LC_50_ against HepG2 than against HaCat cells. Even though amidation increases the peptide cytotoxicity, the selectivity of AamAP1-Lys for pathogens over mammalian cells improves (Tables 2/3).

**Table 3:** Selectivity Index Determined for a Panel of Susceptible and Resistant Bacterial Pathogens and Two Human Cell Lines

	selectivity index (HepG2)^a^			selectivity index (HaCat)^a^		
bacteria	AamAP1-Lys	AamAP1-Lys-NH_2_	fold increase	AamAP1-Lys	AamAP1-Lys-NH_2_	fold increase
*E. coli* ATCC 700928	12.8	6.6		31.1	25.3	
*E. coli* ATCC 25922	1.5	2.9	2	3.6	11.3	3
*P. aeruginosa* ATCC 27853	0.4	0.8	2	0.9	2.9	3
*A. baumannii* ATCC 19606	3.0	6.6	2	7.3	25.3	3
*K. pneumoniae* ATCC BAA-1705	0.7	1.6	2	1.8	6.0	3
*E. cloacae* ATCC 700323	1.5	2.9	2	3.6	11.3	3
*A. baumannii* NCTC 13302	1.5	3.1	2	3.6	11.7	3
*A. baumannii* NICD 15126	1.5	3.1	2	3.6	11.7	3
*A. baumannii* NICD 15207	1.5	3.1	2	3.6	11.7	3
*A. baumannii* NICD 15282	2.9	3.1	0	7.2	11.7	∼2
*A. baumannii* NICD 15283	1.5	3.1	2	3.6	11.7	3
*A. baumannii* NICD 15408	2.9	3.1	0	7.2	11.7	∼2

a The selectivity indexes were determined using the LC_50_ for HepG2 and HaCat cells.

For most of the pathogens, amidation induces a 2-fold or 3-fold increase in the SI over HepG2 cells and HaCat cells, respectively. The only exceptions are *E. coli* ATCC 700928 for which AamAP1-Lys has a higher SI than AamAP1-Lys-NH_2_ over HepG2 and HaCat cells. Overall, the SI values show that AamAP1-Lys-NH_2_ has better selectivity than AamAP1-Lys. Furthermore, the improvement in the SIs due to amidation are larger relative to HaCat cells (2- or 3-fold improvement) compared to HepG2 cells (0- or 2-fold improvement). Therefore, AamAP1-Lys-NH_2_ may have greater potential for development as a topical antibacterial or antibiofilm agent, specifically against *A. baumannii* infections, for which the selectivity indexes are all >10, relative to HaCat cells.

### Amidation Improves Preventative and Inhibitory Action, but not the Eradication of Biofilms

Due to the high SIs against *E. coli* and *A. baumannii* planktonic cells, the ability of AamAP1-Lys and AamAP1-Lys-NH_2_ to eradicate (MBEC), inhibit (MBIC) and prevent (MBPC) biofilms was determined for *E. coli* ATCC 700928 and resistant *A. baumannii* NICD 15283 (Table 4). The selected strains represent the bacteria against which both AMPs are the most active and have the highest SIs (*E. coli* ATCC 700928) (Table 3), as well as a resistant clinical isolate (*A. baumannii* NICD 15283).

**Table 4:** Antibiofilm Activity of AamAP1-Lys and AamAP1-Lys-NH_2_ against Gram-Negative Bacterial Strains

	*E. coli* ATCC 700928		*A. baumannii* NICD 15283	
antibiofilm activity^a^	AamAP1-Lys	AamAP1-Lys-NH_2_	AamAP1-Lys	AamAP1-Lys-NH_2_
MBPC	320 (148)	160 (74)	320–640 (148–296)	**160–**320 (**74–**148)
MBIC	640–1280 (296–592)	320 (148)	320–640 (148–296)	**160–**320 (**74–**148)
MBEC	2560 (592)	2560 (592)	1280 (592)	1280 (592)

a Modal MBPC (Minimum Biofilm Prevention Concentration), MBIC (Minimum Biofilm Inhibition Concentration), and MBEC (Minimum Biofilm Eradication Concentration) given in μg/mL and in the corresponding μM given in parentheses; bold values indicate improvement in antibiofilm activity of AamAP1-Lys-NH_2;_ MBPC and MBEC experiments performed in duplicate with each peptide tested in duplicate (*n* = 4); MBIC experiments performed in triplicate with each peptide tested in duplicate (*n* = 6)

Both peptides are able to prevent, inhibit and eradicate biofilms of *E. coli* and *A. baumannii*. For *E. coli* ATCC 700928, the MBPC (Table 4) for AamAP1-Lys and AamAP1-Lys-NH_2_ is 320 μg/mL and 160 μg/mL, respectively. Whereas, against *A. baumannii* NICD 15283 biofilms, the MBPCs for AamAP1-Lys and AamAP1-Lys-NH_2_ increase and is 320/640 μg/mL and 160/320 μg/mL, respectively. The MBICs for AamAP1-Lys are 640/1280 μg/mL and 320/640 μg/mL against *E. coli* ATCC 700928 and *A. baumannii* NICD 15283 biofilms, respectively. For AamAP1-Lys-NH_2,_ the MBICs improve and are 320/640 μg/mL and 160/320 μg/mL against *E. coli* ATCC 700928 and *A. baumannii* NICD 15283 biofilms, respectively. The decrease in both the MBPC and MBIC of AamAP1-Lys-NH_2_ compared to AamAP1-Lys indicates that amidation enhances the ability of AamAP1-Lys to prevent bacterial colonization and biofilm formation in both *E. coli* and *A. baumannii*, as well as its capacity to inhibit the growth of mature biofilms. Although both peptides are able to eradicate biofilms, very high concentrations of AamAP1-Lys and AamAP1-Lys-NH_2_ are required. The MBEC of both AMPs (Table 4) against both *E. coli* ATCC 700928 and *A. baumannii* NICD 15283 biofilms are 2.56 and 1.28 mg/mL, respectively, indicating that no improvement is observed in the MBECs because of carboxy-amidation.

The antibiotic resistant *A. baumanniiNICD* 15283 biofilms are more readily eradicated and inhibited by both AMPs compared with *E. coli* ATCC 700928. In addition, AamAP1-Lys-NH_2_ prevents and inhibits both types of biofilms more readily, indicating that although both AMPs effectively prevent and inhibit *A. baumannii* biofilms, carboxy-amidation of AamAP1-Lys increased antibiofilm activity.

### Amidation Offers Larval Protection in an *In VivoG. mellonella* Burn Wound Model

**Figure 1:**
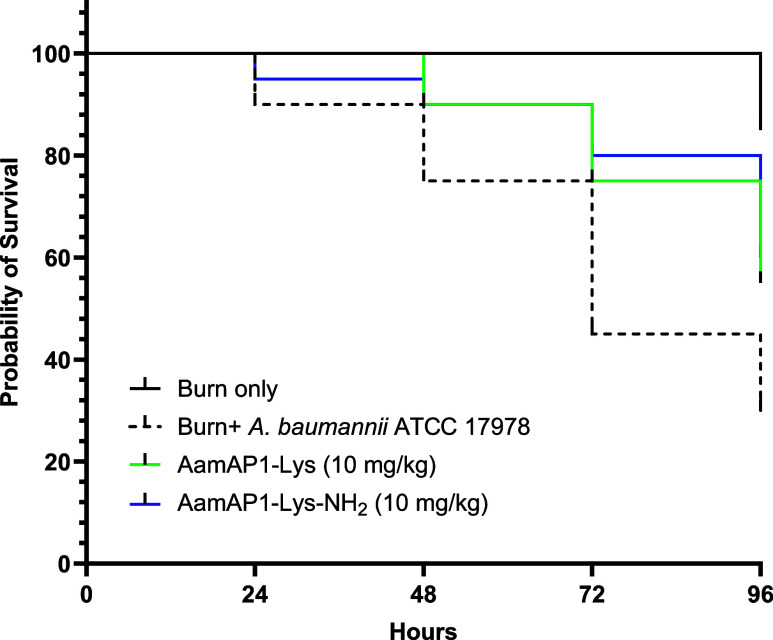
AMPs protect *G. mellonella* larvae with burn wounds infected with *A. baumannii* ATCC 17978. Survival curves are plotted for 20 larvae treated with AamAP1-Lys or AamAP1-Lys-NH_2_ at a single dose of 10 mg/kg compared with 20 larvae subjected to a burn only or burn plus infection with *A. baumannii* ATCC 17978 over 96 h. Percentage survival and the significance of protection against infection due to therapy, according to Log-rank (Mantel–Cox) and Gehan-Breslow-Wilcoxon test are summarized in Table S1.

The high SI for *A. baumannii* relative to HaCat cells and better anti-*A. baumannii* biofilm activity prompted further evaluation of both peptides for *in vivo* protection of *G. mellonella* larvae in a burn wound infection model (Figure 1).

Treatment with AamAP1-Lys increases % survival 4 days postinfection from 30 to 55% (*p* = 0.0645, Mantel-Cox test) but AamAP1-Lys-NH_2_ performs slightly better, increasing survival from 30 to 60% (*p* = 0.0358, Mantel-Cox test) (Figure 1 and Table S1). These results are consistent with carboxy-amidation related gains in potency and selectivity modestly improving the potential for treatment of topical *A. baumannii* infections.

### Amidation Alters Secondary Structure, Conformational Flexibility, Aggregation Patterns, Membrane Area, and Increases Membrane Insertion

To better understand the gains in antibacterial potency, selectivity and therapeutic potential we then conducted a biophysical study focusing on the membrane interaction of the two peptides. First, the secondary structures of AamAP1-Lys and AamAP1-Lys-NH_2_ were investigated using circular dichroism (CD) spectroscopy in Tris buffer, in anionic SDS micelles and in simple models of Gram-negative bacteria plasma membranes composed of POPE/POPG (75:25 mol/mol) liposomes (Figure S1). The CD spectra obtained of both AamAP1-Lys and AamAP1-Lys-NH_2_ are consistent with previous spectra obtained in the same membrane-mimicking environments by our group.^19^ In both SDS micelles and in POPE/POPG (75:25 mol/mol) liposomes both peptides have spectra that are characteristic of α-helix conformation although the characteristic negative bands (208 and 222 nm) are more intense for AamAP1-Lys-NH_2_ possibly indicating a slightly more ordered structure for the amidated analog.

**Figure 2:**
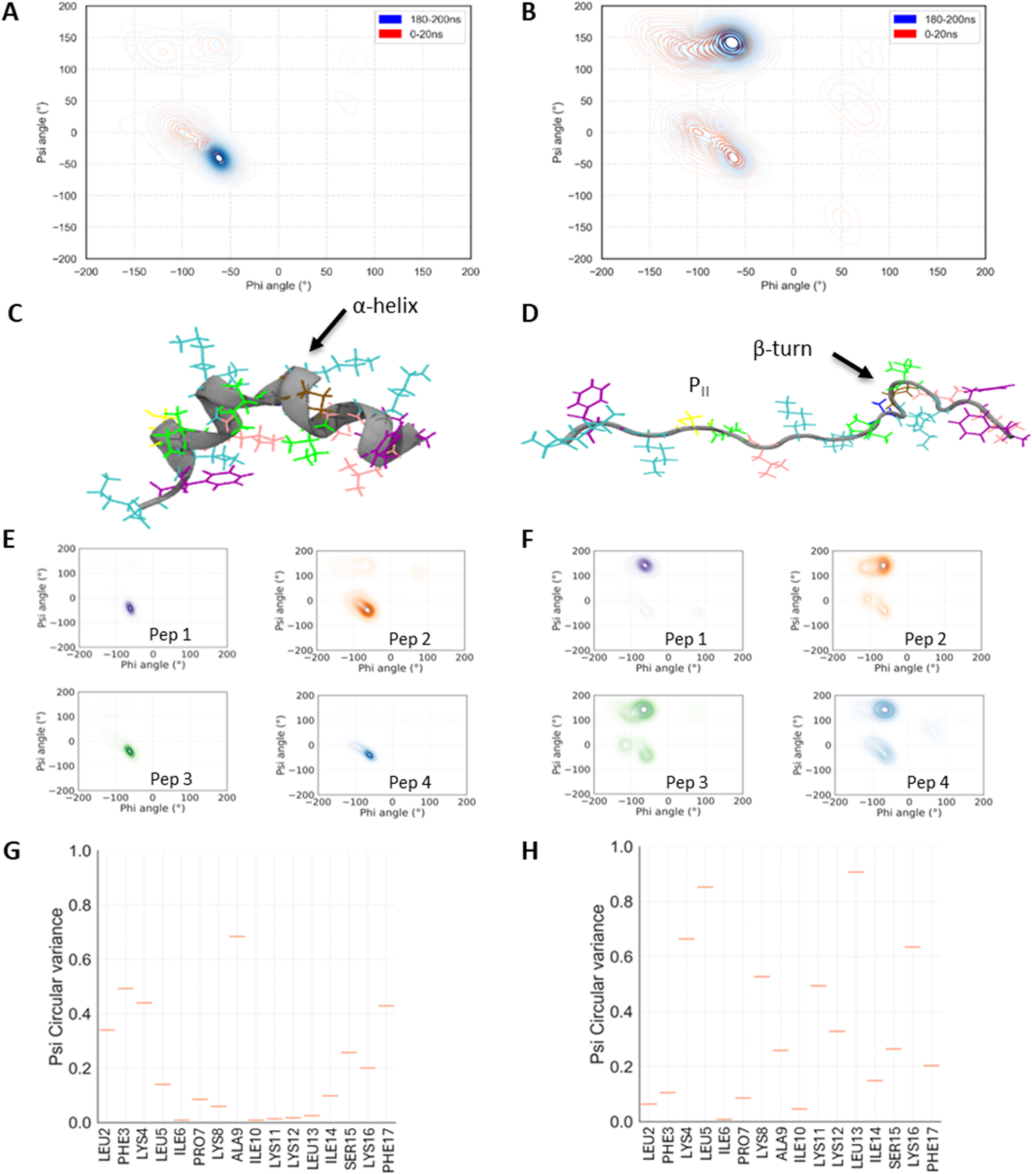
Amidation increases the conformational flexibility of AamAP1-Lys. The secondary structures and circular variance of the AMPs upon interaction with a Gram-negative bacterial PEPG (POPE/POPG, 3:1 ratio) model membrane during the first replicate MD simulation. The Ramachandran contour plots of (A) AamAP1-Lys and (B) AamAP1-Lys-NH_2_ shows the clustering of dihedral angles during 0–20 ns (red) and 180–200 ns (blue) of the simulations. Snapshots of representative (C) AamAP1-Lys and (D) AamAP1-Lys-NH_2_ at the end of the simulation show the α-helix, β-turn, and P_II_ conformations. Residues Ala (blue), Phe (purple), Lys (cyan), Ile (green), Leu (pink), Ser (yellow), and Pro (ochre) are shown. The Ramachandran plots of individual peptides (pep1–pep4) were constructed for (E) AamAP1-Lys and (F) AamAP1-Lys-NH_2_ to evaluate the secondary structures of each individual peptide during the last 80 ns of the simulations. The circular variances of the psi angles of (G) AamAP1-Lys and (H) AamAP1-Lys-NH_2_ are given as a measure of conformational flexibility and over the duration of the simulation and averaged across four peptides and indicate the amount of variance (low = rigid, high = flexible) within the dihedral angles of each residue.

To investigate whether the initial secondary structure and conformational flexibility upon membrane interaction differs between the two AMPs and from the secondary structure determined from the CD analysis obtained in the steady state, we performed MD simulations monitoring the initial 200 ns of the peptide interaction with a model bilayer. Here, the psi and phi dihedral angles that AamAP1-Lys and AamAP1-Lys-NH_2_ adopt upon interaction with Gram-negative bacterial PEPG (POPE/POPG, 3:1 ratio) model membranes were plotted on Ramachandran contour plots (Figure 2A–F/S2A–F).^58,59^ Alongside the secondary structures, the conformational flexibilities of the peptides were evaluated by determining the circular variance of the psi dihedral angles of each residue in each peptide (Figure 2G,H/S2G,H).

In previous work using the same methodology we have identified α-helix conformation for temporin L and a mixture of α-helix and polyproline II for pleurocidin and other peptides from the Winter Flounder.^37,60^ Here there are notable differences in the conformational behavior of the two peptides during the initial binding and (attempted) insertion into the bilayer. AamAP1-Lys primarily adopts an α-helix conformation (Figures 2A,C,E/S2A,C,E) and this is consistent with the typical α-helical CD spectrum in the POPE/POPG (75:25 mol/mol) liposomes observed in the steady state (Figure S1A).

In contrast, although CD spectra obtained for AamAP1-Lys-NH_2_ are similar to those for AamAP1-Lys in both SDS and POPE/POPG (75:25 mol/mol) liposomes in the steady state, MD simulations reveal that amidation causes a reduction of α-helix conformation, with some evidence of type I β-turn (φi+1 −60°, ψi+1 −30°, φi+2 −90°, ψi+2 0°), but the appearance of substantial polyproline II (φ −75°, ψ 150°) (Figures 2B,D,F/S2B,D,F). In both replicate MD simulations, only one of eight AamAP1-Lys peptides (pep2) adopts both polyproline II and α-helix conformations over the last 80 ns of the simulation (Figure S2E). The remaining seven AamAP1-Lys peptides are exclusively found in an α-helix conformation (Figures 2E/S2E). In contrast, the peptides in the replicate MD simulations of AamAP1-lys-NH_2_ adopt both polyproline II and α-helix conformations during the equivalent period (Figures 2F/S2F). This is reflected in the circular variance analysis which reveals that AamAP1-Lys is characterized by higher conformational rigidity (low circular variance), particularly central residues (Ile6-Lys8 and Ile10-Leu13) (Figures 2G/S2G). The N- and C-terminal residues exhibit greater flexibility. In contrast, AamAP1-Lys-NH_2_ is characterized by increased conformational flexibility (high circular variance) in the central region, with fewer residues exhibiting rigidity compared with AamAP1-Lys (Figures 2H/S2H).

Overall, the MD analysis of secondary structures and conformational flexibilities qualitatively correlates with the CD spectra. AamAP1-Lys and AamAP1-Lys-NH_2_ both tend to adopt α-helical structures. However, MD is able to distinguish differences in the two peptides behavior on initial membrane binding: the α-helicity of AamAP1-Lys is more frequent and rigid, while AamAP1-Lys-NH_2_ initially exhibits substantial polyproline II conformations in addition to α-helices with improved flexibility, especially in the central region of its sequence. These insights contribute to understanding the structural dynamics and functional properties of the peptides in the context of Gram-negative bacterial membranes.

Understanding the mechanism of action of AMPs requires determining their propensity for self-association and the residues involved in driving interpeptide interactions. Due to the variability observed in our duplicate MD simulations, we refrained from specifying a single dominant aggregate form for each peptide and chose a more cautious approach. In the case of AamAP1-Lys and AamAP1-Lys-NH_2_, both peptides readily form dimers and higher-order aggregates (Figures S3A–F/S4A–F) such as trimers and tetramers during the simulations, and these may be essential for antimicrobial activity. A snapshot of the aggregates formed by AamAP1-Lys and AamAP1-Lys-NH_2_ at 200 ns shows the association of the nonpolar residues between the individual peptides (Figures S3C,D/S4C,D).

AamAP1-Lys aggregates are formed with interactions between nonpolar residues across the length of the peptide, including N- and C-terminus residues (Figures S3E/S4E). This indicates a more distributed involvement of residues in interpeptide interactions. On the other hand, AamAP1-Lys-NH_2_ aggregates are predominantly formed with interactions between N-terminus nonpolar residues, with no involvement of C-terminus residues, specifically Phe17 and Lys18 (Figures S3F/S4F). This suggests a more specific interaction involving the N-terminus of AamAP1-Lys-NH_2_ in aggregate formation. These differences in residue involvement in aggregation between AamAP1-Lys and AamAP1-Lys-NH_2_ highlight distinct structural differences, that may contribute to differences in the conformational flexibility and thus the membrane interaction.

**Figure 3:**
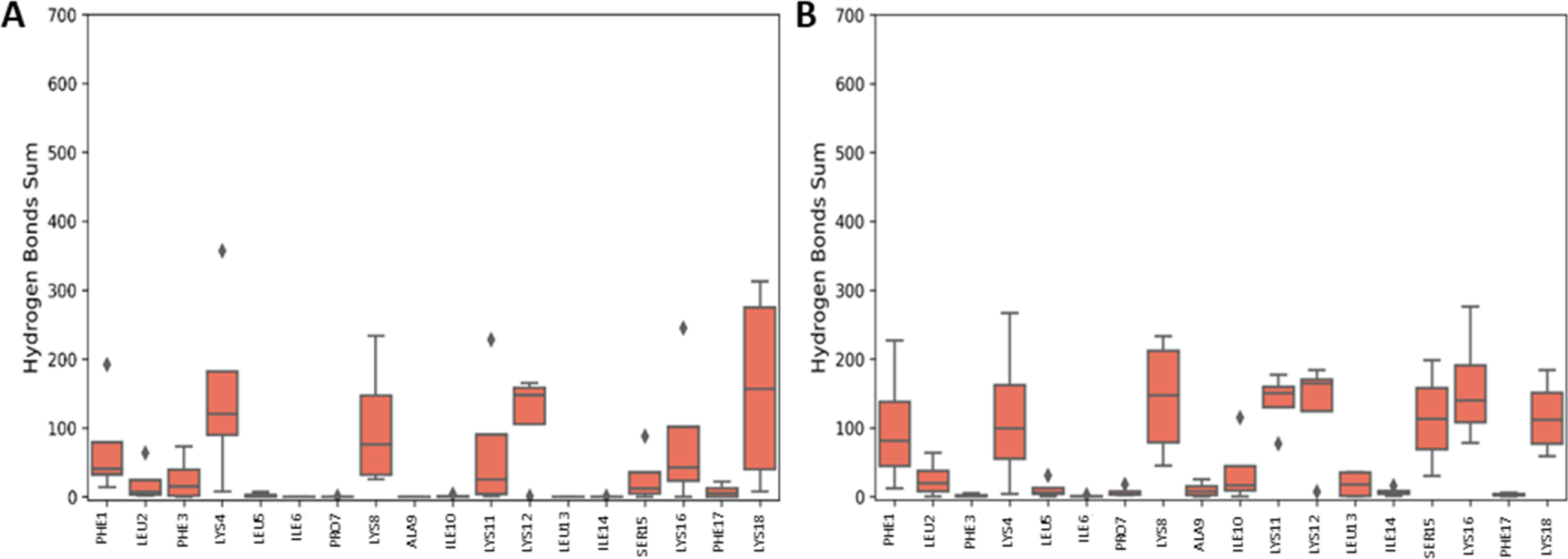
Amidation increases hydrogen bonding of the C-terminus Ser residue with the model membrane. The total amount of hydrogen bonds formed between the AMPs and the lipid headgroups of the Gram-negative bacterial PEPG (POPE/POPG, 3:1 ratio) model membrane during the first replicate MD simulation. The boxplots indicate the sum of hydrogen bonds formed during each simulation for each residue in (A) AamAP1-Lys and (B) AamAP1-Lys-NH_2_. The sums are averaged across four peptides and over the entire 200 ns simulations.

The extent to which the AMPs interact with the Gram-negative bacterial PEPG (POPE/POPG, 3:1 ratio) model membranes was evaluated by determining the number of hydrogen bonds that form between each peptide residue and the model membrane lipid headgroups over the duration of each MD simulation (Figures 3A,B/S5A,B). AamAP1-Lys and AamAP1-Lys NH_2_ form hydrogen bonds predominantly between all their lysine (Lys4, Lys8, Lys16, and Lys18) residues and the lipid headgroups of the Gram-negative bacterial model membranes. Many hydrogen bonds also form between 1Phe, in both peptides, and the lipid headgroups of the model membrane. The π-ring system of 1Phe in both AMPs can act as a hydrogen bond donor.^61^ Fewer hydrogen bonds form between the serine residue (Ser15) of AamAP1-Lys and the membranes compared with AamAP1- Lys-NH_2_. This difference may be attributed to the increased neutral C-terminal interaction with model membrane observed for AamAP1-Lys-NH_2_.

**Figure 4:**
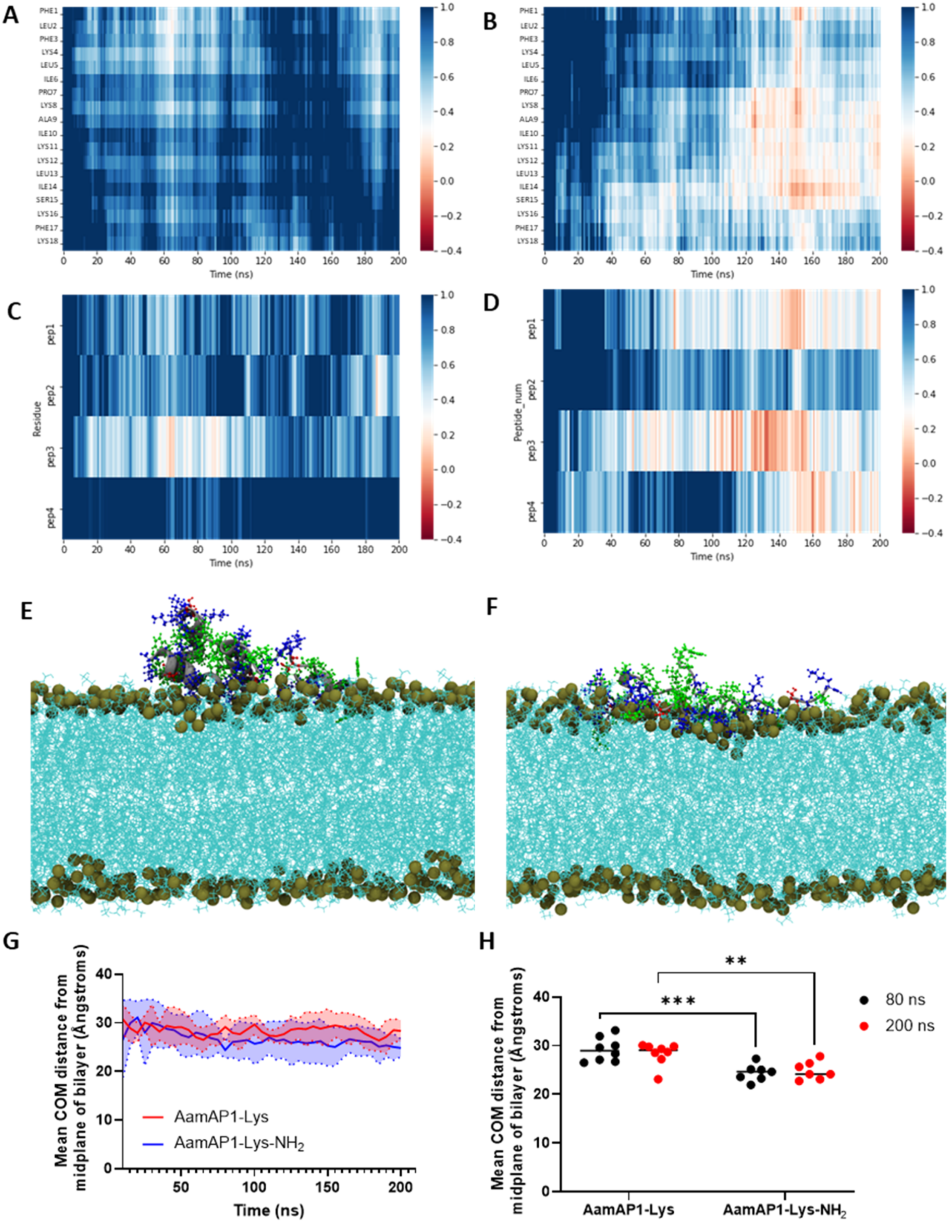
Amidation improves the penetration into model membranes. The insertion of the AMPs into a Gram-negative PEPG (POPE/POPG, 3:1 ratio) model membrane during the first replicate 200 ns MD simulation. Relative *z*-position values above and below 0.4 nm indicate that the α-carbons of the residues/peptide number are not inserting (blue, light blue, and white) or inserting (orange red) into the phosphate group plane, respectively. The relative *z*-positions (nm) of the residues of (A) AamAP1-Lys and (B) AamAP1-Lys-NH_2_ are presented as heatmaps. The average *z*-position of each peptide number for (C) AamAP1-Lys and (D) AamAP1-Lys-NH_2_ are shown as heatmaps to better evaluate individual peptide number insertion during the simulation. The insertion of the (E) AamAP1-Lys and (F) AamAP1-Lys-NH_2_ are shown as VMD snapshots at 200 ns to better visualize their membrane interactions. At 200 ns AamAP1-Lys peptides forms a tetramer and AamAP1-Lys-NH_2_ forms a trimer in the membrane. The tan spheres represent the phosphorus atoms of the phosphate group plane. Basic residues are shown in blue, and nonpolar and polar residues shown in green and red, respectively. The lipids are shown in cyan. The approximate distance between the midplane and phosphate group plane is 18.5 Å (1.85 ± 0.17 nm). (G) The mean center of mass (COM) distance of the peptides from the midplane of the bilayer was determined over the course of the simulations. The mean COM was calculated from duplicate MD simulations and averaged across four peptides in each replicate (*n* = 8). The standard deviation (SD) of the mean COM distance is indicated by the shaded areas. (H) Statistical significance between the mean COM at 80 and 200 ns between AamAP1-Lys and AamAP1-Lys-NH_2_ was determined from two-way analysis of variance (ANOVA) with Šídák’s multiple comparisons test. Significance indicated by ** and *** represent *p* < 0.01 and *p* < 0.001.

The ability to bind and penetrate the membranes of their target organism determines the activity of AMPs.^60,62,63^ With duplicate MD simulations, the interaction of both peptides with Gram-negative bacterial PEPG (POPE/POPG, 3:1 ratio) model membranes was determined. The interaction and insertion of AamAP1-Lys and AamAP1-Lys-NH_2_ into Gram-negative bacterial membranes was evaluated using all-atom MD simulations at the same peptide concentration (Figures 4/S6).

The insertion of specific residues into membranes was quantified by calculating the z position of the residue α-carbons relative to the average *z*-position of the phosphate groups in the upper leaflet of the lipid bilayer.^18,36,61^ The criterion for determining if a residue interacts with the membrane is based on the distance between the residue α-carbon and the phosphorus atoms of the lipid headgroups (phosphate group plane) of the membrane. A residue is considered to interact with the phosphate group plane if this distance is less than 6 Å (0.6 nm) and indicates insertion into the membrane interface.^35,64,65^

The analysis conducted on the relative *z*-positions of the residues in each analog reveals distinct membrane interface insertion behaviors for AamAP1-Lys and AamAP1-Lys-NH_2_. Specifically, AamAP1-Lys-NH_2_ exhibits a greater propensity for insertion compared with AamAP1-Lys and inserts into the bacterial membranes via both the N- and C-termini residues (Figures 4B/S6B), as well as centrally located residues (Figure 4B). In contrast, AamAP1-Lys primarily inserts via its C-terminal (Figure S6A), with minimal interaction by the N-terminal residues (Figures 4A/S6A). Amidation of the C-terminal results in a neutral (−NH_2_) rather than negatively charged (−COO^-^) C-terminus, reducing repulsive forces between the peptide and the negatively charged model membrane. Consequently, AamAP1-Lys-NH_2_ exhibits enhanced C-terminus interaction with the membrane, potentially contributing to its antimicrobial activity. The fraction of peptides inserting within the same simulation was assessed (Figures 4C,D/S6C,D). One to two AamAP1-Lys peptides insert briefly while in contrast, three AamAP1-Lys-NH_2_ peptides insert for longer times during the duplicate 200 ns simulations. Overall, AamAP1-Lys-NH_2_ exhibits a higher tendency to insert, and stay inserted, in Gram-negative bacterial model membranes compared with AamAP1-Lys, based on the higher peptide fraction and readily observed insertion behavior of this AMP. A snapshot of the membrane interaction of AamAP1-Lys and AamAP1-Lys-NH_2_ at 200 ns shows the increased insertion of the nonpolar residues of AamAP1-Lys-NH_2_, specifically 1Phe (N-terminus), Leu13 and Ile14 (central) and Phe17 (C-terminus) (Figures 4E,F/S6E,F). To quantify the distance of the peptides from the midplane of the bilayer core, the mean center of mass (COM) distance of the peptides from the midplane was determined from duplicate MD simulations (Figure 4G,H). The mean COM distance of AamAP1-Lys-NH_2_ from the midplane decreases compared to the mean COM distance of AamAP1-Lys from ∼80 ns onward (Figure 4G). At 80 and 200 ns, the mean COM distance of AamAP1-Lys-NH_2_ across duplicate simulations is significantly smaller (*p* = 0.0004 and *p* = 0.0076) than the mean COM distance of AmAP1-Lys from the midplane, and indicates better binding/penetration into the membrane interface (Figure 4H).

**Figure 5:**
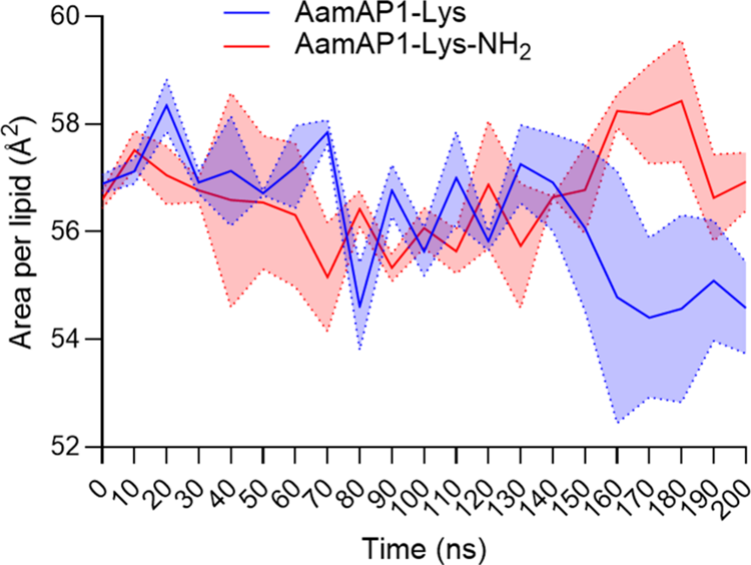
Amidation leads to model membrane stretching upon AMP binding. The change in average area per lipid (APL) of Gram-negative bacterial PEPG (POPE/POPG, 3:1 ratio) model membranes is shown during the course of the simulations. The APL was calculated from duplicate MD simulations and averaged across 256 lipids in each replicate. The standard error of the mean (SEM) is indicated by the shaded areas.

It is well-known that the binding of AMPs to the surface of lipid bilayers, alters the physical properties of the membrane. One of the key effects of this binding is an increase in membrane area, often referred to as membrane “stretching”, which is a critical factor in the formation of membrane pores by AMPs. This stretching is primarily due to the insertion of AMPs into the lipid bilayer, which causes localized changes in the bilayers.^66−68^ The change in area per lipid (APL) induced by binding of AamAP1-Lys and AamAP1-Lys-NH_2_, as a measure of the Gram-negative bacterial model membrane stretching, was examined using our duplicate MD simulations (Figure 5). Substantial changes in APL induced by binding of the AMPs to the model membranes are evident from 140 ns onward. An increase in APL is caused by AamAP1-Lys-NH_2_ between 140 to 180 ns, which indicates membrane stretching. In contrast, AamAP1-Lys decreases the APL from 140 to 200 ns, correlating with membrane compaction.

The orientation of the peptides to the phosphate plane of the membrane over duplicate MD simulations was analyzed to appreciate any differences that could lead to enhanced membrane interface penetration. AamAP1-Lys consistently maintains an angle between 0–20° relative to the phosphate plane throughout the simulation (Figure S7). In contrast, AamAP1-Lys-NH_2_ exhibits an angle between 20–40° during the first 50 ns, after which it stabilizes at a similar angle to AamAP1-Lys, ranging from 0–20°. Although AamAP1-Lys-NH_2_ approaches the membrane at an increased angle, both AamAP1-Lys and AamAp1-Lys-NH_2_ insert into the membrane interface at similar angles.

### Amidation Induces Increased Membrane Permeabilization and Faster Killing of Planktonic Bacterial Cells

**Figure 6:**
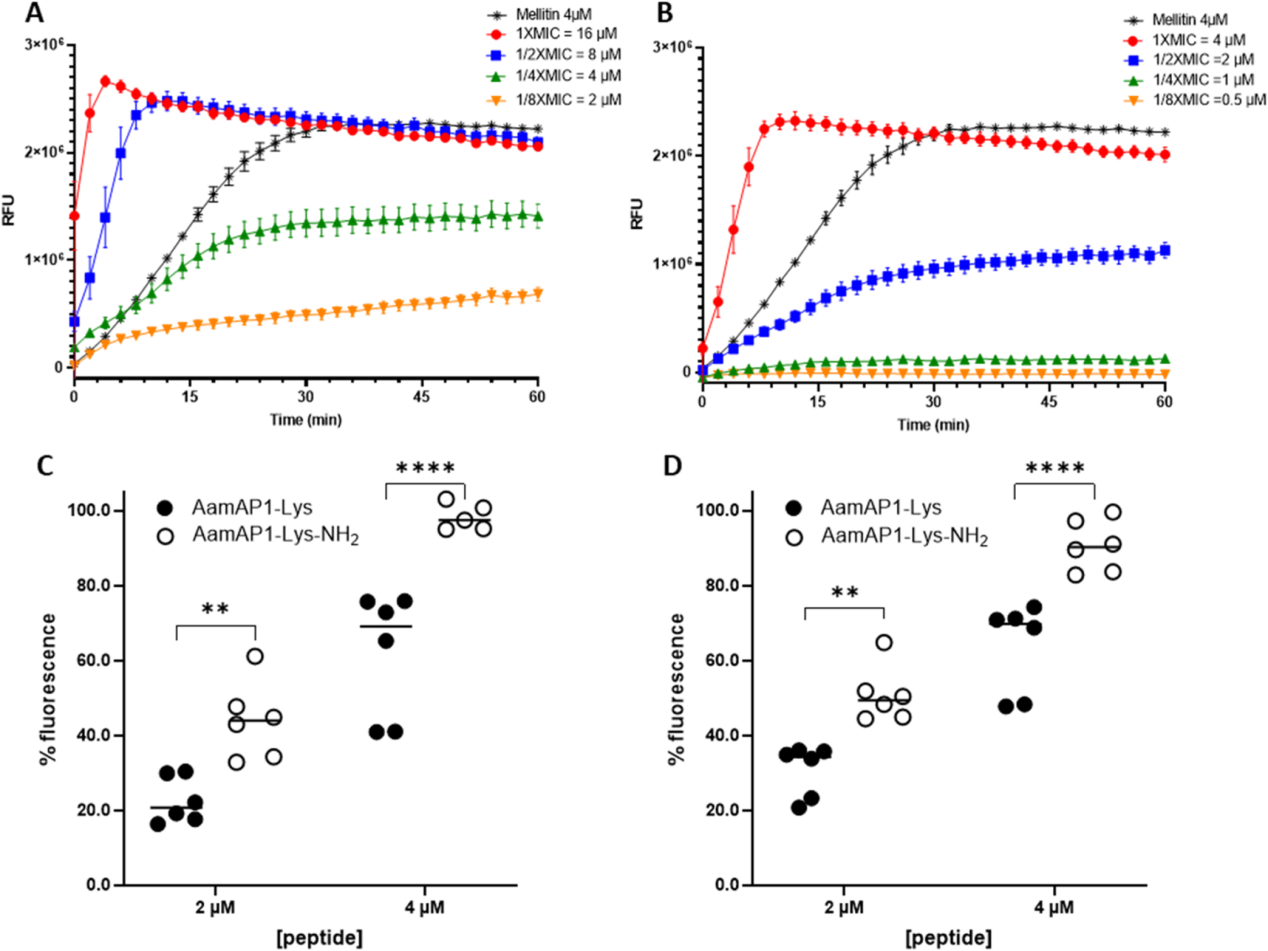
Amidation leads to increased membrane permeabilization and the requirement of a threshold concentration for membrane permeabilization. Inner membrane permeabilization of *A. baumannii* NICD 15283 while incubated with the AMPs. The relative fluorescence units (RFU) were measured for 60 min after addition of (A) AamAP1-Lys and (B) AamAP1-Lys-NH_2_ at concentrations equal to 1× MIC, 1/2× MIC, 1/4× MIC and 1/8× MIC. The % fluorescence after addition of 2 and 4 μM of the peptides at (C) 30 min and (D) 60 min were determined. The membrane permeabilizing peptide, Melittin, was used as a positive control (100% cell damage). Data represent the mean RFU or % fluorescence with SEM from two technical repeats done in triplicate (*n* = 6). Significance indicated by ** and **** represent *p* < 0.01 and *p* < 0.0001 values as determined from two-way ANOVA with Šídák’s multiple comparisons test.

The MD simulations show that amidation impacts the ability of AamAP1-Lys to interact and penetrate the Gram-negative model membranes. Therefore, we sought to investigate the membrane permeabilizing activity of the AMPs using *in vitro* studies. Membrane permeabilization studies using SYTOX green nucleic acid dye were conducted on *A. baumannii* NICD 15283, an antibiotic-resistant clinical isolate, challenged with 0.125, 0.25, 0.5, and 1× MIC of each AMP (Figure 6). Melittin, a lytic peptide and positive control, causes a rapid increase in permeabilization at 4 μM reaching a maximum after 30 min (Figure 6A/B). In contrast, AamAP1-Lys (Figure 6A) and AamAP1-Lys-NH_2_ (Figure 6B) reach a fluorescence maximum after 4 and 10 min, respectively, at their respective MICs of 16 and 4 μM (Figure 6A/B). For both AamAP1-Lys and AamAP1-Lys-NH_2_ the extent of permeabilization is similar to melittin (Figure 6A/B) after 30 min at their 1× MIC. AamAP1-Lys permeabilizes the membrane even at 1/8 of its MIC (Figure 6A). In contrast, for AamAP1-Lys-NH_2_ increased membrane permeabilization only occurs at 1/2 its MIC, indicating the requirements of a threshold concentration for membrane damage to occur (Figure 6B).

At absolute concentrations of 2 and 4 μM the amidated analog, AamAP1-Lys-NH_2_, causes significantly higher (*p* < 0.01, *p* < 0.0001) permeabilization after 30 and 60 min compared with AamAP1-Lys (Figure 6C,D).

**Figure 7:**
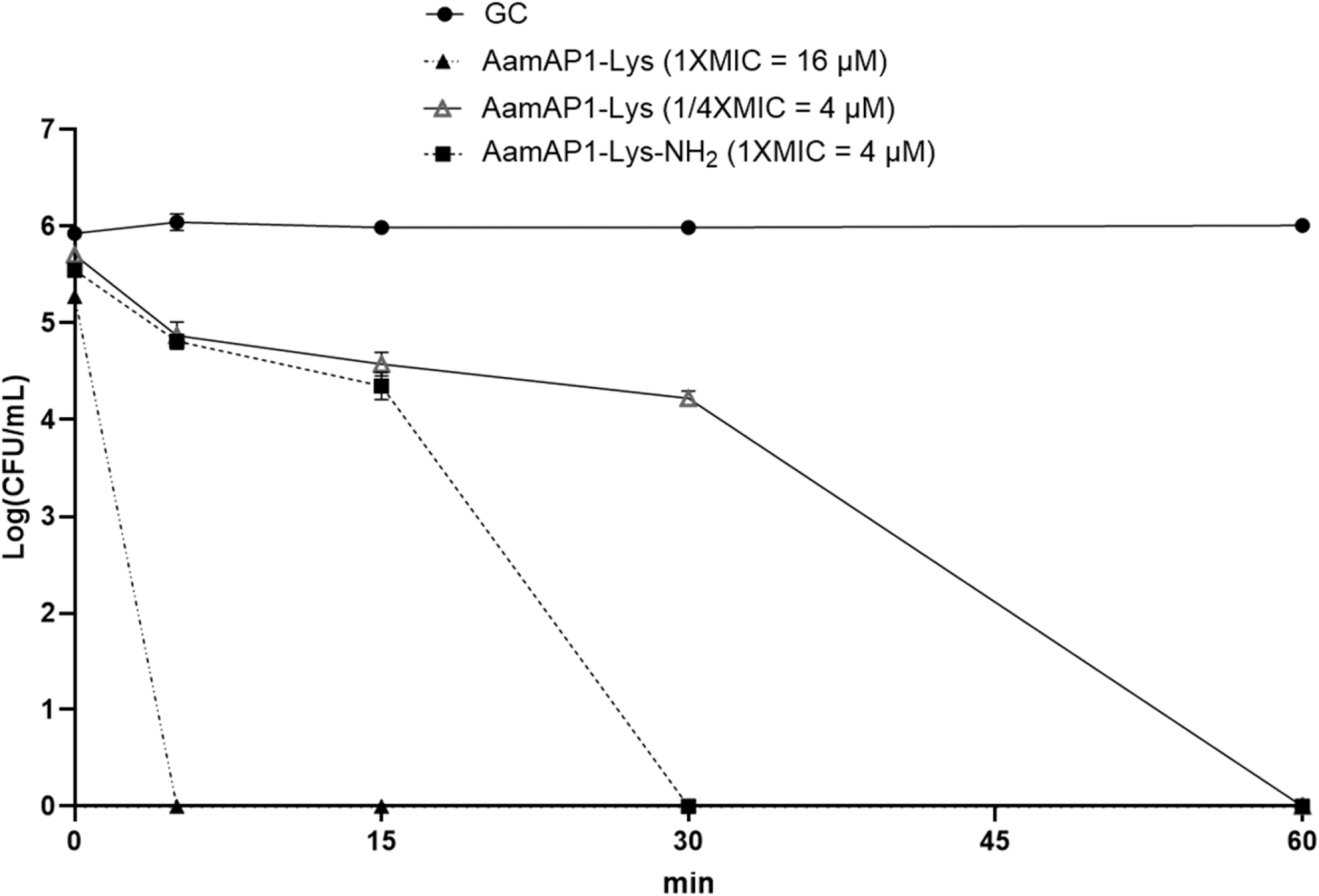
Amidation leads to accelerated bactericidal killing at equal concentrations and corresponding to the MIC AamAP1-Lys-NH_2_. Time-kill kinetics studies of the peptides against *A. baumannii* NICD 15283. The peptides were incubated with the bacterial cells at their respective MICs and at 4 μM for 60 min. Data represented by 2 biological repeats, each done in duplicate (*n* = 4). The growth control contained untreated cells and the Limit of Detection (LOD) is 143 CFU/mL.

To understand whether these differences in time-resolved membrane permeabilization of *A. baumannii* NICD 15283 translate into differences in *in vitro* bacterial killing kinetics, time-kill studies were conducted for AamAP1-Lys and AamAP1-Lys-NH_2_ (Figure 7). At their MICs, AamAP1-Lys causes a 3-log reduction in CFU/mL of *A. baumannii* rapidly within 5 min but the more potent AamAP1-Lys-NH_2_ takes 25 min longer to achieve the same reduction.

However, when the two analogs are compared at the same absolute concentration of 4 μM, the amidated AamAP1-Lys-NH_2_ reduces the CFU/mL below the limit of detection within 30 min, whereas AamAP1-Lys takes 60 min to achieve the same effect. This indicates that, for the same amount of peptide, amidation confers faster and greater permeabilization and faster bacterial killing but that at the new lower MIC, permeabilization and killing is slower than for the less potent parent peptide.

## Discussion

Even though amidation has improved the activity and selectivity of AamAP1-Lys and shows promise for further evaluation in mammalian models due to its protective abilities in the *in vivoG. mellonella* wound model, other pressing questions remain unanswered. These include how does C-terminal amidation affect the structure of AMPs during initial binding to bacterial membranes, specifically Gram-negative membranes, and how does this relate to membrane penetration *in silico* and to what extent does this affect *in vitro* permeabilization, speed of killing bacterial cells and ultimately the AMP pharmacodynamic profile.

Here we find that amidation has significant effects on key aspects of the structure–activity relationship of AamAP1-Lys. First, upon membrane interaction, amidation induces flexible heterogenic conformations consisting of polyproline II, alongside α-helix exclusively found for AamAP1-Lys, and this correlates with improved membrane penetration and antibacterial activity. This is linked to faster and increased plasma membrane permeabilization by AamAP1-Lys-NH_2_, and faster killing of bacteria at equal concentrations of the AMPs. However, at the lower MIC of AamAP1-Lys-NH_2_, permeabilization is slower and less and killing of bacteria is slower, related to a critical concentration for membrane permeabilization to occur.

### Time-Resolved and Steady-State Methods Offer Different Perspectives on Binding of AMPs to Membranes

It has been observed frequently that increased secondary structure stability is associated with improved antibacterial activity of amidated AMPs. The amidation of mastoparan polycationic peptide, protonectarina-MP-ONH_2_, was conclusively shown to induce stabilization of the α-helix with higher α-helical content present determined from CD spectra and molecular modeling.^69^ Similarly, amidated modelin-5-CONH_2_ had higher % helicity compared with the free acid modelin-5-COOH as determined from the steady state CD spectra of the AMPs in *E. coli* lipid extract.^28^ In agreement with this previous work, we also find that CD spectroscopy, in both anionic micelles and simple models of bacterial plasma membrane, suggests a slight increase in ordered α-helix conformation. This may be interpreted as stabilizing secondary structures, through amidation, causing maximization of the presumed membrane disruptive conformation that target membranes.

While CD spectroscopy is again proven to be a robust method of analyzing secondary structure in more complex environments, it is less suited to studying the initial insertion of AMPs into their target membranes, about which much less is known and which may also be critical to antibacterial activity. Indeed the activity of AMPs has been proposed to follow a two-step mechanism in which the first step is membrane association, followed by helix formation and membrane penetration/translocation.^28^ Unfortunately, due to the time scale and the nature of the methods, biophysical studies such as CD analysis are not sensitive to these steps and interactions. An addition to steady-state analysis of AMP secondary structures is all-atom MD simulations analysis, which can better explain the initial binding of AMPs at an atomistic level - in the present study during the first 200 ns of peptide-membrane interactions. Critically, MD simulations are not restricted to reporting on average parameters but can instead reveal different conformations and membrane interaction behavior for individual peptides within a larger population and how this can be affected by minor modifications.

### MD Simulations Enable Identification of Heterogeneity in AMP Membrane Interactions

Our previous work highlighted the effect that composition of model membranes can have on the secondary structures of AMPs when analyzed with CD spectroscopy, with many AMPs changing conformation depending on the type of bacterial model membrane representative used.^19^ Furthermore, Cheng et al. emphasized that the lipid composition and ratios are crucial factors when studying AMP interactions with membranes. The lipid composition of the inner membrane in *E. coli* and other Gram-negative bacteria can vary depending on the strain and environmental conditions, but certain patterns are commonly observed.^70^ In general, the typical lipid composition of the *E. coli* membrane is considered to be approximately 75% POPE, 20% POPG, and 5% cardiolipin (CL).^71,72^ When using simplified models of Gram-negative bacterial membranes, a commonly accepted lipid composition in model systems is the POPE/POPG (3:1) ratio (75% zwitterionic lipids: 25% anionic lipids).^73^ Using compositions that closely resemble the target membrane of interest (in this case, Gram-negative bacterial membranes) is essential for accurate MD simulation results.^74^

For instance, CD and MD simulation analysis revealed that in general, the amidated analogs of aurein 2.9-COOH and aurein 3.1-COOH have greater tendencies to adopt α-helical structures in representative bacterial and mammalian model membranes consisting of either trifluoroethanol (TFE), 1 dimyristoylphosphatidylcholine (DMPC) or dimyristoylphosphatidylserine lipids (DMPS).^33^ However, depending on the membrane mimicking environment, different conformational trends were seen between the free acid forms (aurein 3.1-COOH and aurein 2.6-COOH) and their amidated analogs (aurein 3.1-CONH2 and aurein 2.6-CONH2). Overall, the amidated analogs had a higher tendency to adopt α-helical structures in the presence of the TFE, DMPS, and DMPC, according to CD and MD simulation analysis. However, when inserted into the mammalian representative DMPC membranes, the nonamidated analogs adopted increased α-helical content. This is in contrast to increased α-helical content observed from MD simulation analysis for the amidated analogs of aurein 3.1-COOH and aurein 2.6-COOH in the TFE and DMPS model membranes, highlighting the effect membrane composition can have on secondary structures of AMPs.

Although the above-mentioned study has investigated effects of C-terminal amidation of a set of AMPs on membrane interaction using MD simulations,^33^ model membranes representing mammalian membranes^33^ or general bacterial membranes were used which do not represent Gram-negative membranes exclusively. Therefore, there is a scarcity of detailed atomistic-level simulations that focus on amidated AMPs specifically in Gram-negative and not Gram-positive bacterial or mammalian model membranes. Nevertheless, there are interesting parallels between the present study, where we observe somewhat different behaviors for individual AMPs within each simulation, and a study by Dennison et al. of maximin H5,^29^ even if the membrane models differ. In agreement with previous studies, CD spectroscopy indicated that the more active C-terminal amidated maximin H5 (MH5N) has increased α-helix content relative to the native AMP (MH5C) in the presence of zwitterionic DMPC lipids. The corresponding MD simulations in DMPC model membranes, however revealed that only one of the three MH5N peptides in the simulation adopts an α-helix and this was the MH5N peptide primarily responsible for membrane penetration during the MD simulation. The remaining two MH5N peptides were unfolded during the MD simulations. Both this previous work and the present study therefore show that while steady-state methods may report increased conformational order, MD simulations reveal that at early timesteps a population of AMPs may adopt a range of different conformations. This heterogeneity in membrane interaction may prove to have important functional impacts.

### Polyproline II and Superior Conformationally Flexibility Induced by Amidation is Revealed as the Key Driver for Better Gram-Negative Model Membrane Penetration

To the best of our knowledge, it has not been reported before that C-terminal amidation reduces initial secondary structure rigidity through induction of polyproline II conformations while antimicrobial activity is substantially improved.

However, less ordered insertion orientation has been revealed in a recent study to be linked to the improved membrane insertion of C-terminal amidated CM15 (CM15-Am) compared to the nonamidated form using MD simulations.^32^ The change in the orientation of insertion from perpendicular to oblique after amidation was identified as the main change implicated in the more disordered insertion orientation of CM15-Am into bacterial model membranes, and is not due an increase in the conformational flexibility.^32^

As a specific effect of C-terminal amidation, increased conformational flexibility during initial membrane binding within a population of AMPs is a new concept and it may not be immediately clear how this leads to improved antibacterial activity. However, greater conformational flexibility has been reported in proline rich AMPs^75^ and in pleurocidin and its analogs^36,61^ and this is associated with increased antibacterial activity. In similar MD simulation studies, when compared to magainin 2, pleurocidin showed greater conformational flexibility, improved membrane insertion and greater antibacterial potency.^64^ Furthermore, the pleurocidin analog, pleurocidin KR, with greater conformational flexibility and antibacterial potency than pleurocidin VA, further supports the notion that conformational flexibility is a key driver in AMP membrane interaction.^36^

A more recent study on ionenes found similarly that conformational flexibility can modulate the antimicrobial activity of synthetic mimics of antimicrobial peptides (SMAMPs). The main-chain flexibility of these cationic polymers were particularly important for improved antimicrobial activity and faster killing kinetics against Gram-negative bacteria (*E. coli*), whereas against Gram-positive bacteria (*Staphylococcus aureus*) only the ionene analogs with main-chain flexibility and the highest hydrophobicity (presence of C8 and C12 alkyl chains) were more active compared to their counterparts (<C8 alkyl chains).^76^

In many instances enhanced antimicrobial activity correlates with larger cytotoxic effects therefore selectivity is unchanged, however, increased selectivity is the desired outcome when designing improved AMPs. Encouragingly Nam et al. showed reducing the helicity of peptoid 1 (fully α-helical) produced the equally active peptoid 17, with improved selectivity compared to the fully α helical peptoid 1 and was attributed to the increased conformational flexibility of peptoid 17.^77^

Therefore, consistent with the studies mentioned above, the gain in potency and selectivity of the amidated AamAP1-Lys derivative can be attributed to increased Gram-negative membrane interaction and permeabilization as a result of increased flexibility and heterogeneity in the conformations adopted compared with the deamidated analog, AamAP1-Lys. The mechanism by which this occurs is as yet unclear but one may speculate that increased flexibility enables an antimicrobial to benefit from a greater number and variety of interactions with different parts of the lipid molecules as it moves through the different strata of the bilayer. The implication of greater conformational flexibility, induced membrane stretching and reduced hydrophobicity of AamAP1-Lys-NH_2_ in pore formation must also be considered, as pore formation is an important mechanism of many AMPs.^66,67^ The stretching of the membrane caused by AamAP1-Lys-NH_2_ may be involved in the formation of a prepore, as a result of decreased lateral density in lipid membranes, which can lead to increased rate of pore formation, in contrast to AamAP1-Lys.^66,78,79^

Greater conformational flexibility may facilitate the stabilization of toroidal pores, as the peptide is better able to bend and adapt to the curved, catenoid shape of the pore rim. Furthermore, peptide flexibility also entropically favors the solution state and decreases adsorption of the peptide to the membrane.^80^ Therefore we speculate that in contrast to AamAP1-Lys, the more conformationally flexible AamAP1-Lys-NH_2_, with its greater affinity for the solution state (evidenced by the shorter RP-HPLC elution time and decreased hydrophobicity)^19^ and binding induced membrane stretching may better support the rapid formation of toroidal pores possibly leading to better antibacterial activity.

### Induction of Polyproline II Structures due to C-Terminal Amidation Remains Unclear

It is not yet clear how C-terminal amidation induces polyproline II structures in AamAP1-Lys but at least two possibilities are suggested by previous studies. The first possibility is that increased membrane interaction at the C-terminus impedes α-helix stabilization. The primary amide has been identified as a specific membrane binding moiety of C-terminal amidated AMPs in a study on aurein1.2.^81^ Changing the primary amide to secondary amide of aurein1.2. resulted in loss of activity and it was concluded that C-terminal amidation is a crucial membrane binding moiety. Similarly, we find that there is more hydrogen bonding between the C-terminal Ser15 residue of AamAP1-Lys-NH_2_ and the model membrane, and indicates better binding of the C-terminus compared with the deaminated AamAP1-Lys. It may be that the binding of the primary amine and/or the overall increased membrane interaction of AamAP1-Lys-NH_2_ interferes with rapid helix formation and initial stabilization. Alternatively or in addition, there may be an interplay between conformational flexibility and aggregation. A machine learning and MD simulation study^35^ revealed that aggregate formation between synergistic AMPs from the same origin can modulate the number of distinct conformations, and thus the conformational flexibility, that the AMPs adopt when in aggregate states.

Analogously, if amidation modifies aggregate formation this has the potential to modulate the conformational heterogeneity and flexibilities of AamAP1-Lys. While such an investigation is beyond the scope of the present study and no clear difference is observed in the tendency and sizes of aggregates formed by the AMPs, a difference is apparent in the residues/areas implicated in aggregate formation. We find that the residues involved in aggregate formation by AamAP1-Lys are nonspecific whereas for the amidated AamAP1-Lys-NH_2_ aggregates are formed between the N-termini residues predominantly, with reduced, or sometimes no, interaction between C-terminus residues. This in turn may reduce the constraint on the conformational space of AamAP1-Lys-NH_2_, consequently leading to conformational heterogeneity and increased presence of polyproline II conformations, in contrast to AamAP1-Lys.

While the MD simulations are as yet unable to identify behavior, such as membrane damage or translocation, that would unambiguously produce a bactericidal effect they nevertheless do show that increased conformational flexibility resulting from amidation is associated with greater penetration of the membrane during initial binding. This in turn would be expected to impact membrane permeabilization and potentially bactericidal kinetics if the former is a key aspect of the bactericidal mechanism.

### Amidation Changes the Pharmacodynamic Profile of AamAP1-Lys

To target the plasma membrane of Gram-negative bacteria, AMPs have first to pass through the outer membrane and the present study is limited because the interactions of the AMPs beyond bacterial plasma membranes were not investigated. Nevertheless, comparison with a similar study may help understand the mechanism of action of AamAP1-Lys. Studies on the interaction of PMAP-23C (free acid) and its amidated derivative PMAP-23N, on lipopolysaccharide (LPS), the major component present in the outer membrane of Gram-negative bacteria, indicated that both AMPs have LPS neutralizing abilities.^31^ However, PMAP-23C and PMAP-23N do not stay associated with LPS and translocate to the inner membrane of the Gram-negative bacteria. Killing kinetics studies of PMAP-23C and PMAP-23N at equal concentrations of 8 μM (2× MIC) using *E. coli* are consistent with our results, showing that amidation accelerates bactericidal killing and is linked to faster and more permeabilization of membranes when both PMAP-23C and PMAP-23N are applied at equal concentrations. Since the killing kinetics of AamAP1-Lys and AamAP1-Lys-NH_2_ are comparable to PMAP-23N and PMAP-23C, it is likely that AamAP1-Lys and its amidated analog similarly translocate to the inner membrane where they exert their action on the plasma membranes. The high membrane permeabilization abilities of AamAP1-Lys and AamAP1-Lys-NH_2_ therefore supports the suggestion that the primary mechanism of action of these AMPs are on the plasma membranes and not the outer membrane of Gram-negative bacteria. Interestingly however the finding here that the gain in potency through amidation of AamAP1-Lys is accompanied by slower killing and reduced and slower permeabilization of bacteria at an improved (lower) MIC indicates that such a small modification can alter the pharmacodynamic profile of an AMP which may have further implications for therapeutic outcomes.

### Impact of AamAP1-Lys Amidation on Therapy is Limited

Although there is an improvement in the selectivity of AamAP1-Lys-NH_2_*in vitro*, in more realistic physiological environment and at equal dose in an *in vivoG. mellonella* model, there seems to be only marginal differences between the efficacy of AamAP1-Lys and its amidated derivative. A likely explanation for this discrepancy is the complexity of the physiological conditions present in an acute burn wound infection, which could affect the antimicrobial activity of the peptides.^82^*In vitro* assays often overestimate the efficacy of antimicrobial agents because they do not fully account for the challenges of the *in vivo* environment, such as host immune responses, tissue penetration, and fluctuating physiological conditions, that can influence peptide activity.^83,84^

Moreover, the conditions in the *in vitro* assays differ significantly from those *in vivo*. For example, in the *in vivoG. mellonella* burn wound model, only a single dose of AamAP1-Lys-NH_2_ was tested, which may not have been optimal, either too low or too high, to effectively exploit the rapid bacterial killing kinetics and membrane permeabilization properties.

Since we find that increased potency does not necessarily induce faster killing and does not translate to significant improvement *in vivo* when applied at equal doses of 10 mg/kg, the question then remains unanswered whether either or both of increased potency or faster killing can translate to better therapeutic outcomes. Future *in vivo* studies should include dose response administration of the AMPs, to see whether slower killing but improved potency (lower MIC) and better selectivity at their MICs, or faster killing at equal concentrations is more critical to better therapeutic outcomes. To better capture the therapeutic potential of AamAP1-Lys-NH_2_*in vivo*, optimizing the dosing regimen, possibly through multiple doses or varying concentrations, may be necessary. This will be explored in future studies to more accurately reflect the efficacy of the AMPs under clinically relevant conditions.

## Conclusions

This study provides new insights into the effects that C-terminal amidation of AamAP1-Lys has on the structure–activity relationship. We show that C-terminal amidation results in an analog that is significantly more active against Gram-negative pathogens with improved selectivity, attributed to greater conformational flexibility associated with better initial Gram-negative model membrane insertion, membrane permeabilization, and ultimately faster bacterial killing at equal doses. The gain in potency of the C-terminal amidated AamAP1-Lys however should be considered in the context of an altered pharmacodynamic profile, with less membrane permeabilization and slower bacterial killing observed at the lower MIC which is related to a required critical concentration. This altered pharmacodynamic profile may impact the success of AamAP1-Lys-NH_2_ application as a therapy for *A. baumannii* infected burn wounds. Nevertheless, here we find that C-terminal amidation, a minor modification, has substantial impact on AMP conformational flexibility upon initial bacterial membrane interaction and is identified as a possible role player for an altered pharmacodynamic profile.

## Supporting Information Available

Statistical analysis of *in vivo**G. mellonella* burn wound model data, CD spectra of the peptides, results from replicate MD simulations of the peptides in model membranes, and the MICs of conventional antibiotics against five clinical isolates of *A. baumannii* (PDF)
