# Supplementary material for: Carboxy-Amidated AamAP1-Lys has Superior Conformational Flexibility and Accelerated Killing of Gram-Negative Bacteria
Source: Biochemistry. 2025 Jan 28;64(4):841–59. doi: 10.1021/acs.biochem.4c00580 (PMC11840929; doi:10.1021/acs.biochem.4c00580)
Supplement: Supplementary file 1 — bi4c00580_si_001.pdf [file bi4c00580_si_001.pdf]

Supporting information

Carboxy-amidated AamAP1-Lys has superior conformational flexibility and accelerated killing of Gram-negative bacteria

Rosalind J. Van Wyk[a], June C. Serem[b], Carel B. Oosthuizen[c], Dorothy Semenya[d], Miruna Serian[e], Christian D. Lorenz[f], A. James Mason[d*], Megan J. Bester[b] and Anabella R. M. Gaspar[a*]

[a] Department of Biochemistry, Genetics and Microbiology, Faculty of Natural and Agricultural Sciences, University of Pretoria, Pretoria, 0002, South Africa

[b] Department of Anatomy, Faculty of Health Sciences, University of Pretoria, Pretoria, 0002, South Africa

[c] Drug Discovery and Development Centre (H3D), University of Cape Town, Cape Town, 7701, South Africa

[d] Institute of Pharmaceutical Science, School of Cancer & Pharmaceutical Sciences, King's College London, London, SE1 9NH, United Kingdom

[e] Department of Physics, Faculty of Natural, Mathematical and Engineering Sciences, King's College London, London, WC2R 2LS, United Kingdom

[f] Department of Engineering, Faculty of Natural, Mathematical and Engineering Sciences, King's College London, London, WC2R 2LS, United Kingdom

*Prof. Annabella R. M. Gaspar, Department of Biochemistry, Genetics and Microbiology, Faculty of Natural and Agricultural Sciences, University of Pretoria, Pretoria, 0002, South Africa; Email:annabella.gaspar@up.ac.za

*Prof. A. James Mason, Institute of Pharmaceutical Science, School of Cancer & Pharmaceutical Sciences, King's College London, London, SE1 9NH, United Kingdom; Email:james.mason@kcl.ac.uk

Table S1. The percentage survival of burn wound infected (*A. baumannii* ATCC 17978) *G. mellonella* larvae on day 4 after AMP treatment.

**Condition**	**Survival % @ 96 hrs**	**P-value (P > 0.05)**	
		**Log-rank (Mantel-Cox) test**	**Gehan-Breslow-Wilcoxon test**
**Burn only**	85	0.0001	<0.0001
**Burn + Infection**	30	-	-
**AamAP1-Lys (10 mg/kg)**	55	0.0645	0.0522
**AamAP1-Lys-NH_2(10 mg/kg)**	60	0.0358*	0.0321*

Data represents 2 biological repeats consisting of 10 larvae per repeat.
***Values in bold indicate significant differences between the burn and infection control.**

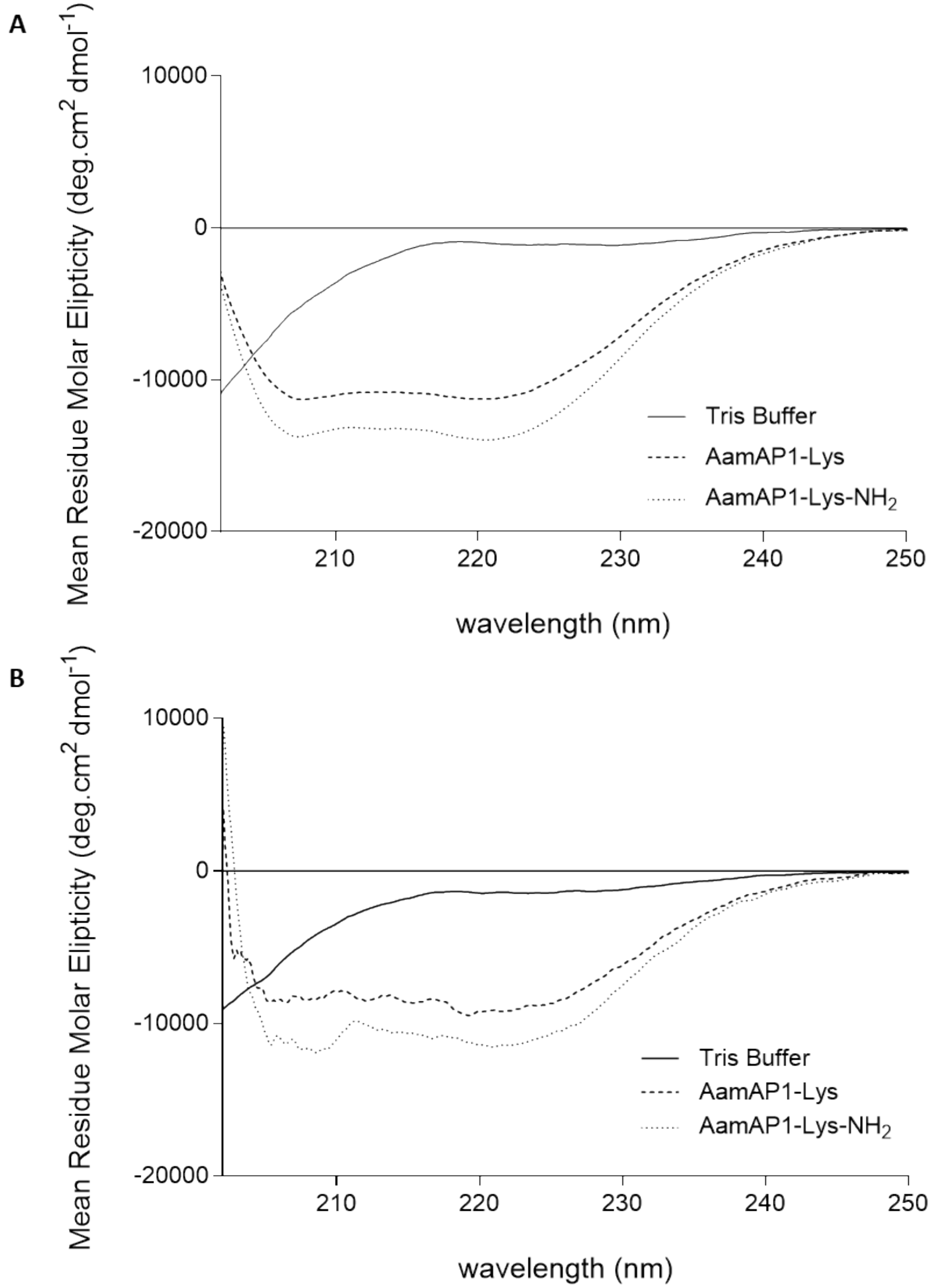

Figure S1. The CD spectra between 202 – 250 nm of AamAP1-Lys and AamAP1-Lys-NH_2 in Tris buffer, A) SDS micelles and B) POPE:POPG (75:25 mol:mol) liposomes.

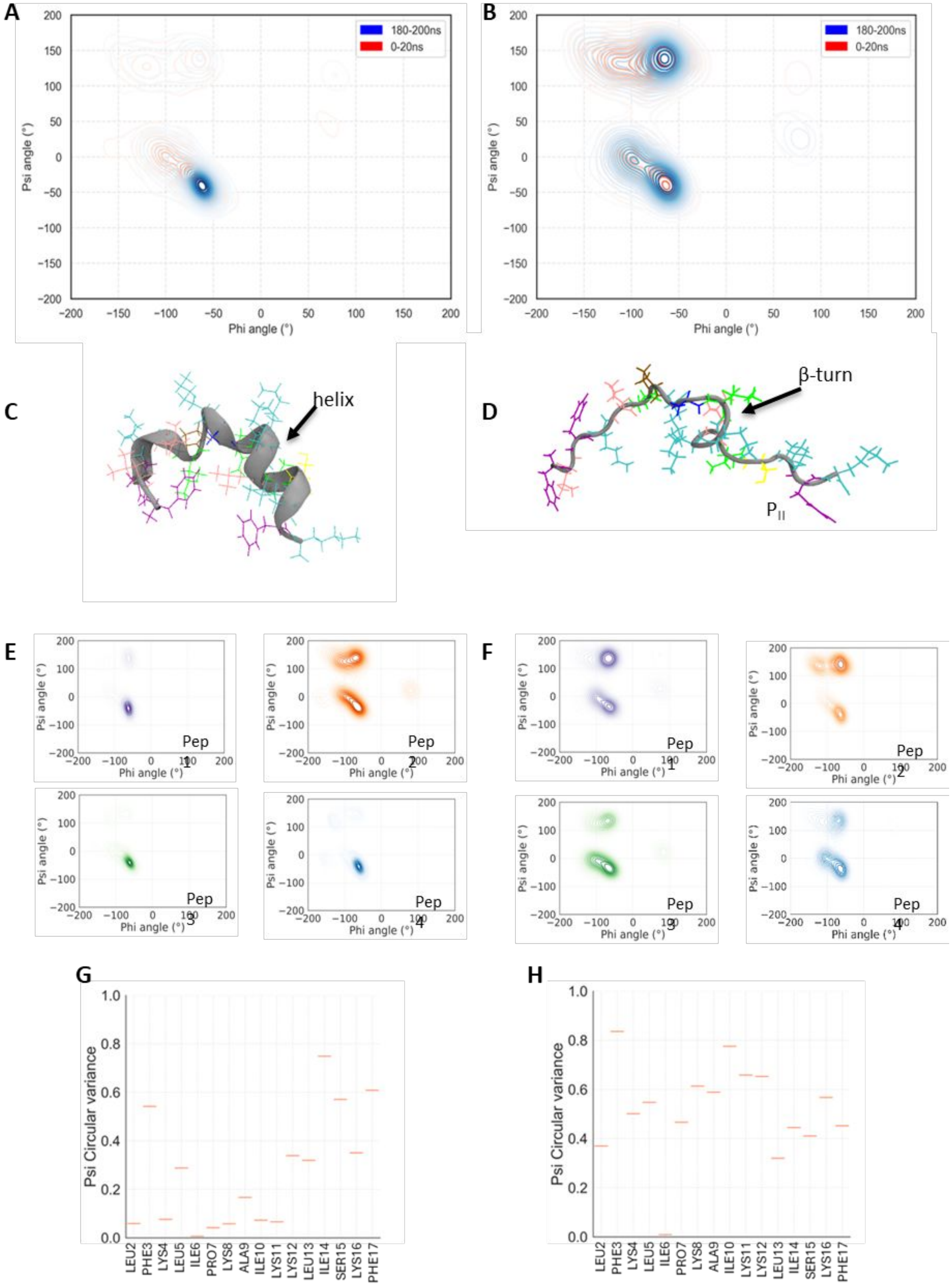

Figure S2. The secondary structures and circular variance of the AMPs upon interaction with a Gram-negative PEPG (POPE:POPG, 3:1 ratio) model membrane during the second replicate MD simulation. The Ramachandran contour plots of A) AamAP1-Lys and B) AamAP1-Lys-NH_2 shows the clustering of dihedral angles during 0-20 ns (red) and 180-

200 ns (blue) of the simulations. Snapshots of representative C) AamAP1-Lys and D) AamAP1-Lys-NH2 at the end of the simulation show the helix, β-turn and (insert d) PPII conformations, indicated by arrows. Residues Ala (blue), Phe (purple), Lys (cyan), Ile (green), Leu (pink), Ser (yellow) and Pro (ochre) are shown. The Ramachandran plots of individual peptides (pep1-pep4) were constructed for E) AamAP1-Lys and F) AamAP1-Lys-NH_2 to evaluate the secondary structures of each individual peptide during the last 80 ns of the simulations. The circular variances of the psi angles of G) AamAP1-Lys and H) AamAP1-Lys-NH_2 are given as a measure of conformational flexibility and over the duration of the simulation and averaged across four peptides and indicate the amount of variance (low = rigid, high = flexible) within the dihedral angles of each residue.

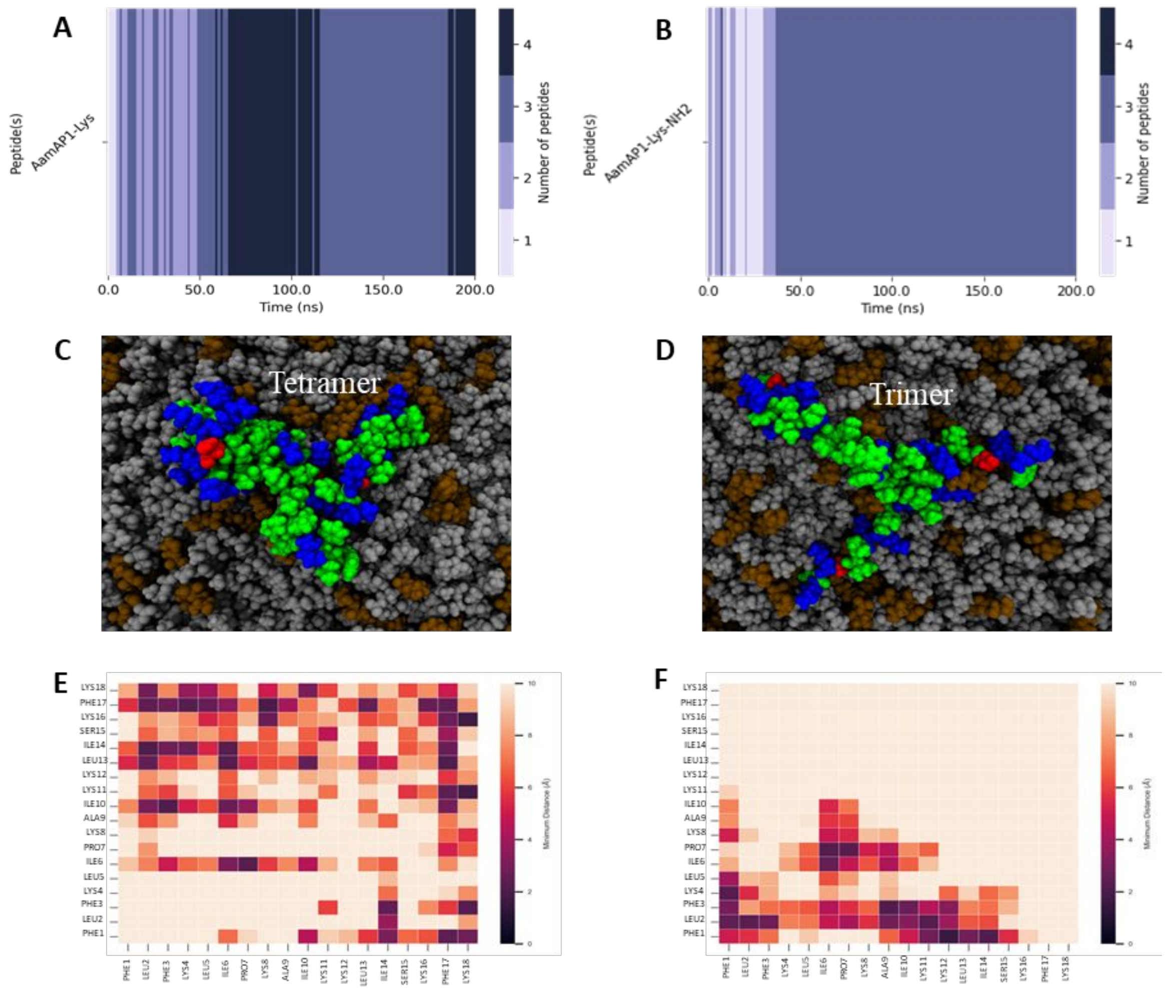

Figure S3. Analysis of the size, stability and implicated residues in aggregates of the AMPs during the first replicate 200 ns MD simulation in a Gram-negative bacterial PEPG (POPE:POPG, 3:1 ratio) model membrane. The size (monomer, dimer, trimer and tetramer) and stability of the aggregates formed by A) AamAP1-Lys and B) AamAP1-Lys-NH_2 are shown as heatmaps. A top view of the C) AamAP1-Lys and D) AamAP1-Lys-NH_2 aggregates in the membranes are shown with basic residues shown in blue, and nonpolar and polar residues shown in green and red, respectively. The POPE and POPG lipids are shown in grey and brown, respectively. The residues that are involved and interacting in the formation of E) AamAP1-Lys and F) AamAP1-Lys-NH_2 aggregates are shown as heatmaps with the distance between the α-carbon of residues indicated by the color bar in Angstrom.

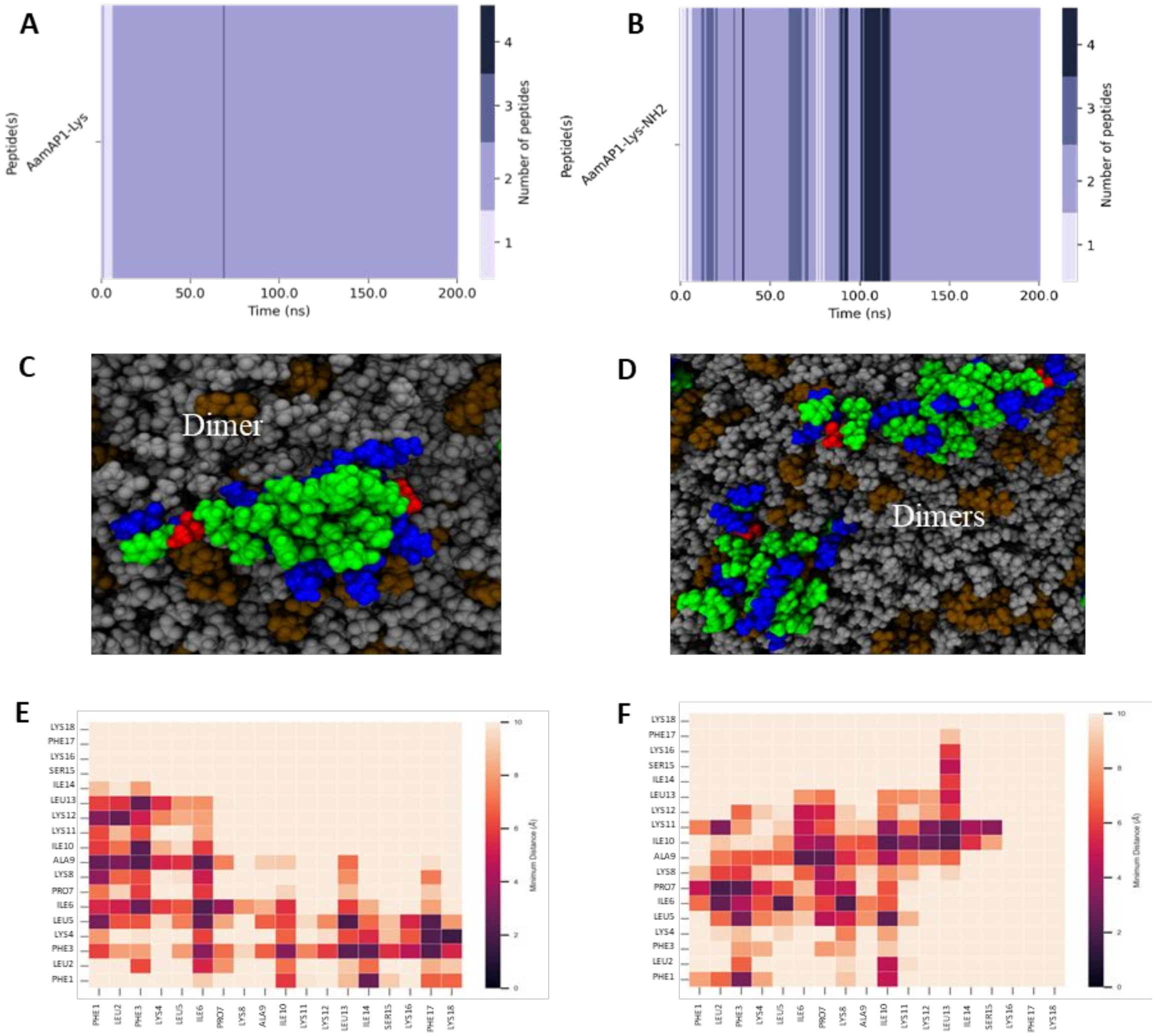

Figure S4. Analysis of the size, stability and implicated residues in aggregates of the AMPs during the second replicate 200 ns MD simulation in a Gram-negative bacterial PEPG (POPE:POPG, 3:1 ratio) model membrane. The size (monomer, dimer, trimer and tetramer) and stability of the aggregates formed by A) AamAP1-Lys and B) AamAP1-Lys-NH_2 are shown as heatmaps. A top view of the C) AamAP1-Lys and D) AamAP1-Lys-NH_2 aggregates are shown with basic residues shown in blue, and nonpolar and polar residues shown in green and red, respectively. The POPE and POPG lipids are shown in grey and brown, respectively. The residues that are involved and interacting in the formation of E) AamAP1-Lys and F) AamAP1-Lys-NH_2 aggregates are shown as heatmaps with the distance between the α-carbon of residues indicated by the color bar in Angstrom.

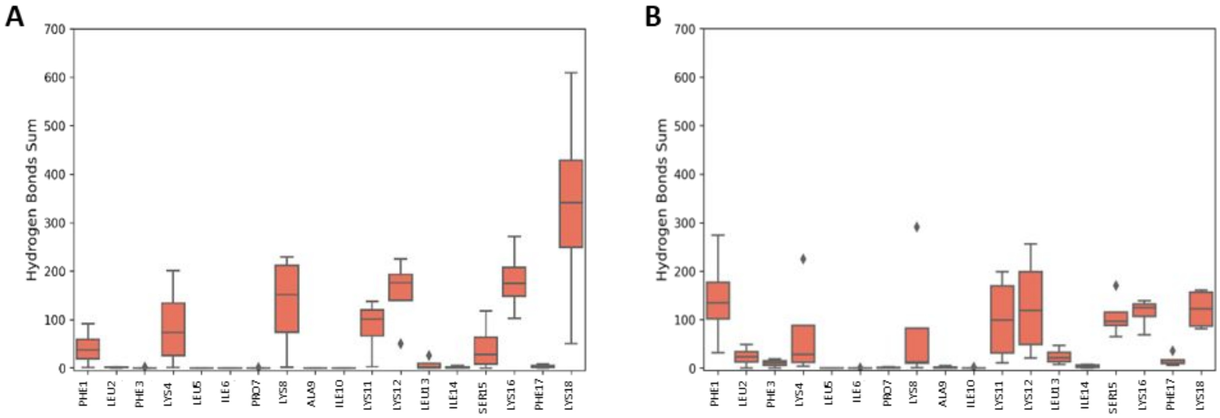

Figure S5. The total amount of hydrogen bonds formed between the AMPs and the lipid headgroups of the Gram-negative bacterial PEPG (POPE:POPG, 3:1 ratio) model membrane during the second replicate MD simulation. The boxplots indicate the sum of hydrogen bonds formed during each simulation for each residue in A) AamAP1-Lys and B) AamAP1-Lys-NH_2. The sums are averaged across four peptides and over the entire 200ns simulations.

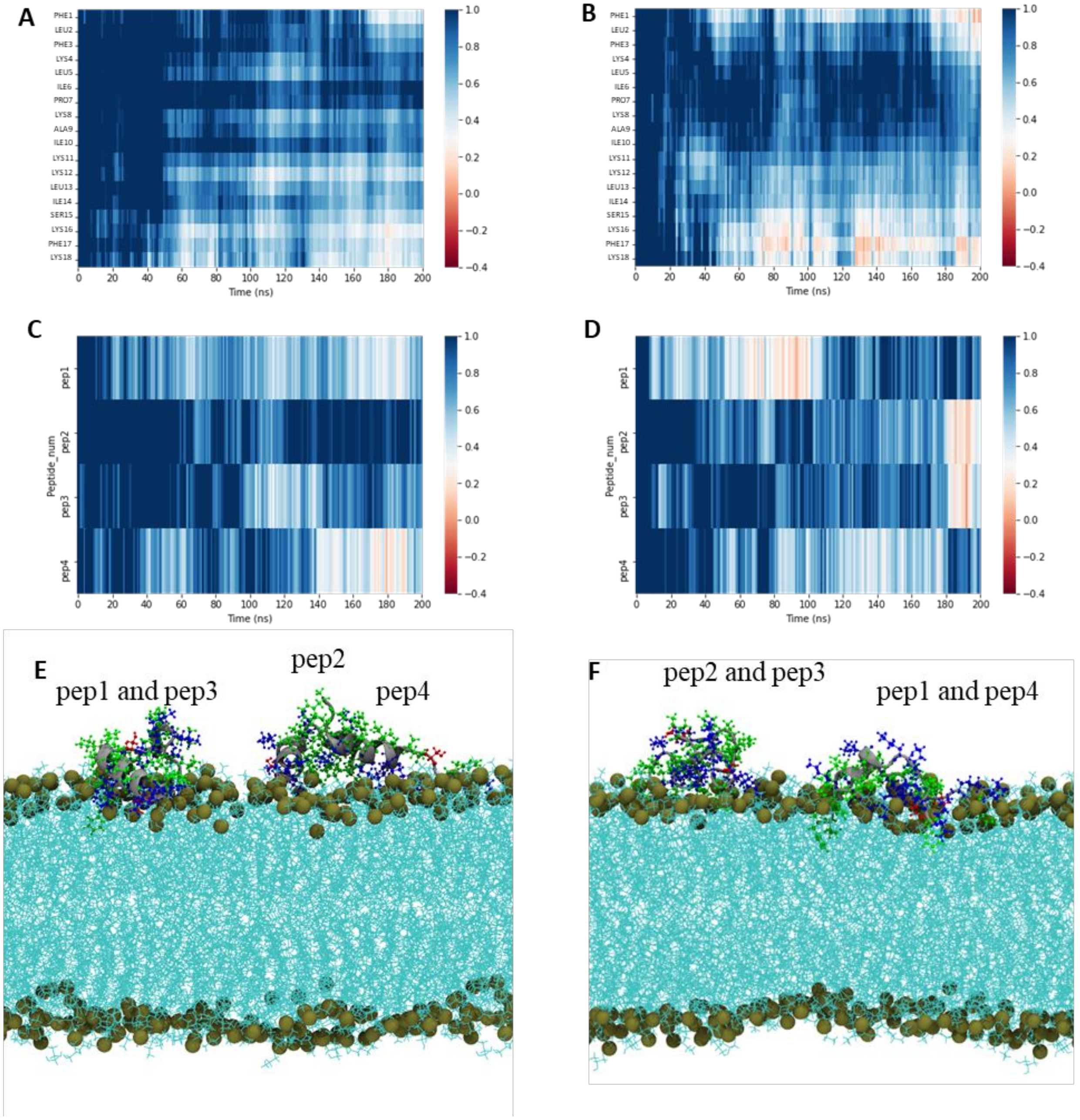

Figure S6. The insertion of the AMPs into a Gram-negative bacterial PEPG (POPE:POPG, 3:1 ratio) model membrane during the second replicate 200ns MD simulation. Relative z-position values above and below 0.4 Angstrom indicate that the α-carbons of the residues/peptide number are not inserting (blue, light blue and white) or inserting (orange red) into the phosphate group plane, respectively. The relative z-positions of the residues of A) AamAP1-Lys and B) AamAP1-Lys-NH_2 are presented as heatmaps. The average z-position of each peptide number for C) AamAP1-Lys and D) AamAP1-Lys-NH_2 are shown as heatmaps to better evaluate individual peptide number insertion during the simulation. The insertion of the E) AamAP1-Lys and F) AamAP1-Lys-NH_2 are shown as VMD snapshots at 200ns to better visualize their membrane interactions. At 200 ns AamAP1-Lys peptides forms a dimer (pep1 and pep 3) and AamAP1-Lys-NH_2 forms dimers (pep1 and pep4, pep2 and pep3) in the membrane. The tan spheres represent the phosphorous atoms of the phosphate group plane. Basic residues are shown in blue, and nonpolar and polar residues shown in green and red, respectively. . The lipids are shown in cyan. The approximate distance between the midplane and phosphate group plane is 18.5 Angstrom (1.85 +-0.17 nm).

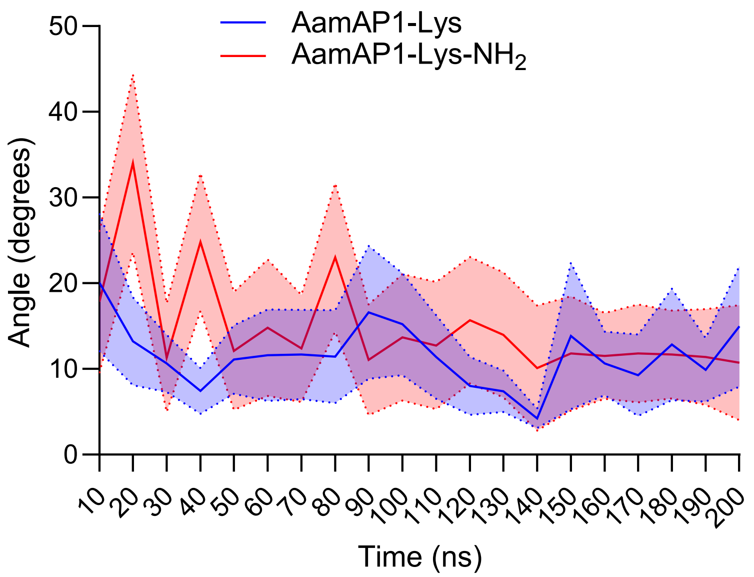

Figure S7. The angle between the AMPs and the membrane phosphate plane during the course of the simulations. The angles of the AMPs (between 0 – 90 °) were calculated and averaged across four AMPs in each simulation (n = 8). The standard error of the mean is indicated by the shaded areas.

Table S2. The MIC of different conventional antibiotics against five *A. baumannii* clinical isolates and one laboratory strain.

***A. baumannii* strain**	**MIC in ug/mL**				
	Polymyxin B	Ciprofloxacin	Moxifloxacin	Meropenem	Colistin
**NCTC 13302 (OXA-25)**	1	>64	32	>64	4
**NICD 15126 (MacS, CarS)**	1	1	0.125	1	4
**NICD 15207 (CarR)**	1	64	32	>64	4
**NICD 15282 (CarR)**	1	>64	64	>64	2
**NICD 15283 (CarR)**	1	1	0.5	32	4
**NICD 15408 (CarR, ColI)**	0.5	>64	64	>64	2

ATCC, American Type Culture Collection – USA; CarR, carbapenem resistant; CarS; carbapenem sensitive; ColI, colistin intermediate; MacS, macrolide sensitive; MIC, minimum inhibitory concentration; NCTC, National Collection of Type Cultures – UK; NICD, National Institute For Communicable Diseases – RSA; OXA-25, OXA-type β-lactamases carbapenem resistance
